# Motor recruitment to the TIM23 channel’s lateral gate restricts polypeptide release into the inner membrane

**DOI:** 10.1038/s41467-018-06492-8

**Published:** 2018-10-02

**Authors:** Alexander Benjamin Schendzielorz, Piotr Bragoszewski, Nataliia Naumenko, Ridhima Gomkale, Christian Schulz, Bernard Guiard, Agnieszka Chacinska, Peter Rehling

**Affiliations:** 1 Department of Cellular Biochemistry, University Medical Center Göttingen, GZMB, 37073 Göttingen, Germany; 2 Göttingen Centre for Molecular Biosciences, Georg-August-University, Göttingen, 37077 Germany; 3 Centre of New Technologies, University of Warsaw, S. Banacha 2c, 02-097 Warsaw, Poland; 4 Centre de Génétique Moléculaire, CNRS, 91190 Gif-sur-Yvette, France; 5 Max Planck Institute for Biophysical Chemistry, 37077 Göttingen, Germany

## Abstract

The presequence translocase of the mitochondrial inner membrane (TIM23 complex) facilitates anterograde precursor transport into the matrix and lateral release of precursors with stop-transfer signal into the membrane (sorting). Sorting requires precursor exit from the translocation channel into the lipid phase through the lateral gate of the TIM23 complex. How the two transport modes are regulated and balanced against each other is unknown. Here we show that the import motor J-protein Pam18, which is essential for matrix import, controls lateral protein release into the lipid bilayer. Constitutively translocase-associated Pam18 obstructs lateral precursor transport. Concomitantly, Mgr2, implicated in precursor quality control, is displaced from the translocase. We conclude that during motor-dependent matrix protein transport, the transmembrane segment of Pam18 closes the lateral gate to promote anterograde polypeptide movement. This finding explains why a motor-free form of the translocase facilitates the lateral movement of precursors with a stop-transfer signal.

## Abstract (web summary)

The mitochondrial TIM23-complex facilitates anterograde precursor transport across the inner membrane into the matrix and lateral release of precursors into the membrane. Here authors show that the import motor J-protein Pam18 controls lateral protein release into the lipid bilayer.

## Introduction

Protein transport machineries of the endoplasmic reticulum, chloroplasts, and mitochondria facilitate anterograde protein translocation across membranes as well as lateral protein transport of precursors with hydrophobic transmembrane regions into the lipid phase. Proteinaceous channels enable passage of the precursors across membranes and provide lateral gates for lipid phase access. In case of mitochondria, the majority of precursors are targeted by N-terminal signals, termed presequences^1,2^. The precursors enter mitochondria through the translocase of the outer membrane (TOM) from which they are handed over to the presequence translocase (TIM23 complex) in the inner membrane^3–6^. The membrane potential (∆ψ) across the inner membrane acts on the positively charged presequence to drive initial translocation of the N terminus through the TIM23 complex^7^. The TIM23 complex facilitates the transport of two basic types of precursors (i) matrix proteins that are fully translocated across the inner membrane and depend on the activity of the ATP-driven import motor for transport; (ii) inner membrane proteins that are stalled during the process of inner membrane translocation and laterally released into the lipid phase^5,6,8–10^. The latter process requires the existence of a lateral gate in the translocation channel that provides access to the lipid phase for the translocating polypeptide chain.

Inner membrane proteins contain a hydrophobic stop-transfer signal, also referred to as sorting signal, that stalls translocation allowing preproteins to be released into the lipid bilayer^11^. The current concept on this transport process by the TIM23 complex postulates that the import channel can open toward the lipid phase by an unknown mechanism to allow precursor exit^12^. The TIM23 complex contains six integral membrane proteins, which could be involved in this process. Pam17 is only transiently associated with the complex and supports import of ∆ψ-sensitive proteins but is especially important for translocation of soluble proteins^13^. Tim50 exposes a receptor domain to the intermembrane space (IMS) and regulates channel opening^14–16^. Tim21 interacts with Tim50 and couples the TIM23 complex to the respiratory chain^16,17^. Tim23 and probably Tim17 form the channel unit of the translocase. While Tim23 also contains a receptor domain and is the main channel constituent, Tim17 plays a regulatory role by recruiting the import motor and regulating gating activity of the channel^18–20^. Mgr2 couples Tim21 to the translocase and has been suggested to act as a quality-control factor for the sorting process^12,21^.

Protein transport into the matrix requires the import motor. The central mtHsp70 chaperone drives proteins into the aqueous matrix at the expense of ATP. The ATPase activity of mtHsp70 is stimulated by the J-protein Pam18, which is recruited to the translocase and regulated by the membrane-associated J-like protein Pam16^22–25^. Tim44 represents the scaffold protein of the motor that recruits mtHsp70 to the import channel^26^. Similar to Pam16, Tim44 is peripherally associated with the membrane^27^. Both interactions with the inner membrane and direct protein–protein interactions with channel components couple the import motor to the translocase^28^. However, Pam18 is the sole motor component that has a membrane-spanning segment and interacts on both sites of the membrane with translocase components. Interestingly, during translocation of a polypeptide, the import motor is not a stable entity but undergoes subunit exchange with Pam18 displaying the strongest dynamics^29^.

The question on whether the observed dynamics of the import motor is critical for the transport process is controversially discussed. Two distinct states of the TIM23 complex have been observed. A Tim21-containing (TIM23^SORT^) form that is motor free and able to insert proteins laterally into the membrane upon reconstitution and a TIM23^MOTOR^ form lacking Tim21, but instead associated with the motor complex to enable matrix translocation^30,31^. However, it remains unknown, if the motor-containing form of the translocase is solely capable of anterograde transport toward the matrix or also lateral transport into the lipid phase.

Here we find that by constitutively associating Pam18 to the TIM23 complex, lateral transport of translocating polypeptides into the lipid phase is compromised. Our analyses show that Pam18 seals the lateral gate when the motor complex drives anterograde protein transport. These findings now explain why the presequence translocase switches between a motor-bound and motor-free states and as to why the motor-free form of the translocase promotes inner membrane sorting. Our findings suggest that translocase complexes, which utilize lateral gates for membrane insertion of precursors, could regulate lateral movement by recruitment of membrane domains of dynamically associating proteins.

## Results

### TIM23 reorganization by constitutive association of Pam18

**Fig. 1:**
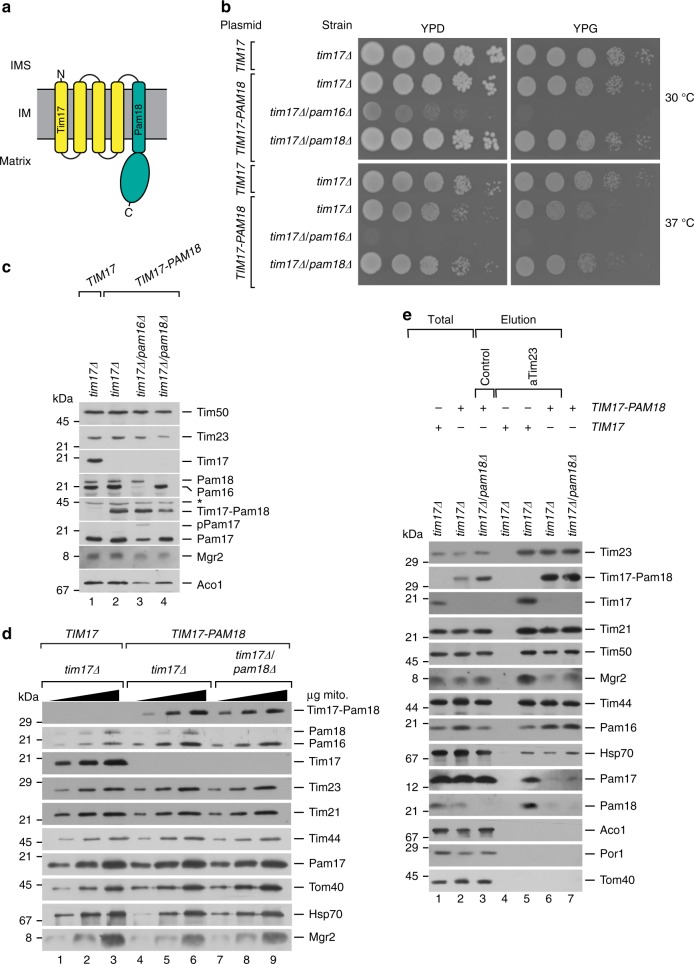
A functional Tim17–Pam18 fusion causes reorganization of the TIM23 complex. **a** Schematic representation of the Tim17–Pam18 fusion. N, N terminus; C, C terminus; IMS, intermembrane space; IM inner membrane. **b** Tenfold serial dilutions of wild-type and indicated Tim17–Pam18 strains were spotted on YPD and YPG plates and grown at indicated temperatures. **c** Western blot analysis of cell lysates from wild-type and Tim17–Pam18 strains. Asterisk, unspecific signal of antibody. Tim17–Pam18 protein was detected using Pam18 antibody. **d** Western blot analysis of steady-state protein using isolated mitochondria (5, 10, and 20 µg). **e** Digitonin-solubilized mitochondria were subjected to anti-Tim23 immunoisolation and samples were analyzed by western blotting. Total, 10%; elution, 100%

Pam18 is the presequence translocase-associated J-protein with a single transmembrane span. Its intermembrane space localized N-terminal domain associates to the translocation channel constituent Tim17. However, Pam18 is not permanently translocase associated but undergoes a replenishment cycle during import. Here we aimed to study the functional relevance of the dynamic association of Pam18 for protein translocation. To this end, we generated yeast strains in which the N terminus of Pam18 was covalently fused to the C terminus of Tim17 in a hybrid protein. Since the intermembrane space exposed C-terminal domain of Tim17 transiently interacts with the N-terminal domain of Pam18, we reasoned that this fusion protein would closely resemble the physiological translocase-associated state (Fig. 1a). In fact, expression of the Tim17–Pam18 fusion protein complemented the lethal growth defect of yeast cells deleted for *TIM17* as well as a *TIM17*/*PAM18* double deletion. The complemented cells displayed only a very mild growth defect on both fermentable and non-fermentable medium (Fig. 1b). Strikingly, expression of the Tim17–Pam18 fusion protein also allowed for deletion of the essential *PAM16* gene. Pam16 is implicated in recruiting and regulating Pam18 at the translocase. These two functions can obviously, at least partially, be overcome by constitutive Pam18 association at the translocase. Nevertheless, these cells displayed a strong growth defect, indicating that a tight control of Pam18 function by Pam16 is crucial for proper import motor function (Fig. 1b). We assessed the expression of the Tim17–Pam18 fusion construct in all tested cells. Importantly, no free Tim17 or Pam18 were detected in the corresponding deletion backgrounds, demonstrating that the fusion construct remained stable in the tested strains (Fig. 1c). Due to the strong growth defect and the apparent import defect in vivo, indicated by the presence of the Pam17 precursor (Fig. 1c, lane 3), the *tim17∆/pam16∆* strain was excluded from further analysis. Most importantly, expression of the Tim17–Pam18 fusion protein did not affect the amounts of other subunits of the translocase or the associated motor in mitochondria (Fig. 1d).

The TIM23 complex can be purified in two forms. The Tim21-containing TIM23^SORT^ form, which lacks the import motor, and the TIM23^MOTOR^ form, lacking Tim21 but that is bound to the motor complex. These two forms are in equilibrium and isolation of the TIM23 complex via the channel-forming Tim23 subunits leads to copurification of both states. As expected, immunoisolation with anti-Tim23 antibodies co-purified the Tim17–Pam18 fusion protein (Fig. 1e). In the context of the Tim17–Pam18 fusion protein, Tim50, Tim44, and Hsp70 co-isolated with Tim23 in wild-type-like amounts. While the amount of Tim21 was slightly reduced, Pam16, which forms a stable complex with Pam18, displayed increased copurification. In contrast, Pam17 and Mgr2 were not efficiently recovered with Tim23, even though their mitochondrial protein levels were not altered (Fig. 1e). Accordingly, covalent association of Pam18 to Tim17 affected the organization of the presequence translocase leading to the loss of the auxiliary subunits Pam17 and Mgr2. Interestingly, free Pam18 was almost completely lost from the TIM23 complex when the C terminus of Tim17 was occupied by the fused Pam18, indicating that this is the primary binding site for Pam18 at the complex. However, the constitutively Pam18-associated translocase must be capable to transport proteins since cells did not display a severe growth phenotype.

### Import motor function of the Pam18-associated translocase

**Fig. 2:**
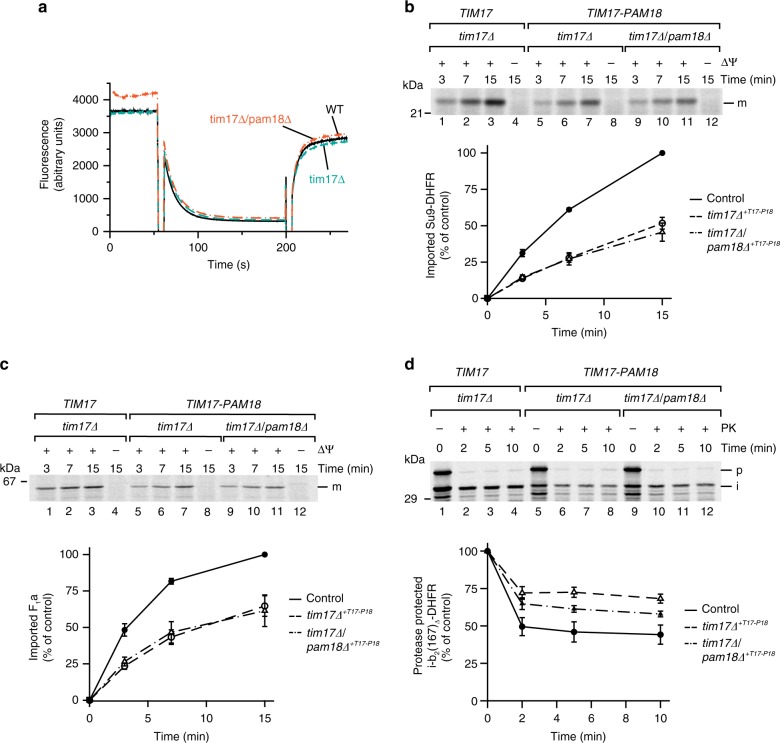
Mitochondria containing a Tim17–Pam18 fusion display mild-matrix import defects. **a** Membrane potential (Δψ) of mitochondria was assessed using the Δψ-sensitive dye DiSC_3_(5). Fluorescence quenching by mitochondria was recorded before and after addition of valinomycin. **b**, **c** [^35^S]-labeled precursor proteins were imported into the mitochondria and import stopped after indicated time points using antimycin A, valinomycin, and oligomycin (AVO). Samples were Proteinase K (PK)-treated and analyzed by SDS-PAGE and digital autoradiography. Results are presented as mean ± SEM, *n* = 3. The longest import time of the WT sample was set to 100%. m, mature protein; *tim17∆*^*+T17-P18*^ *tim17∆* + *TIM17–PAM18*. **d** The inward-driving force of the import motor was assessed using [^35^S]-labeled b_2_(167)_∆_-DHFR in the presence of methotrexate (MTX). After 15 min import, Δψ was dissipated using valinomycin. In a second step, the precursor was chased for indicated time before PK addition. The amount of processed intermediate was quantified (100%: amount of processed intermediate without protease treatment). Results are shown as mean ± SEM, *n* = 4. p, precursor; i, intermediate

Since Pam18 is essential for motor-driven protein import into the matrix^23,25,32^, we aimed to analyze protein transport into mitochondria directly. To this end, we assessed the ∆ψ across the inner membrane that drives the initial presequence translocation. Wild-type and mutant mitochondria containing the Tim17–Pam18 fusion displayed similar magnitudes of ∆ψ (Fig. 2a). Based on the integrity of the ∆ψ, we assessed the matrix import capacity of wild-type and mutant mitochondria by in vitro import analyses. Import of the matrix protein F_1_α (a soluble subunit of the F_1_F_o_-ATP synthase) and the model matrix protein Su9-DHFR were moderately affected in both mutants (Fig. 2b, c). An obvious explanation for this phenotype would be that motor function was partially affected.

Motor activity can be monitored by assessing its inward-driving force with a model matrix protein b_2_(167)_∆_-DHFR. Import in the presence of the substrate analog methotrexate (MTX) prevents translocation of the C-terminal, stable-folded DHFR moiety leading to a translocation intermediate that spans both TOM and TIM23 complexes and engages with the import motor on the matrix side of the inner membrane. Depletion of ∆ψ after precursor accumulation and subsequent proteinase K digestion allows for degradation of the portion of b_2_(167)_∆_-DHFR that is not strongly pulled against the TOM complex by the import motor. Remarkably, mutant strains expressing the Tim17–Pam18 fusion displayed reduced protease-sensitivity indicative of increased motor activity (Fig. 2d). This effect appears to increase with the absence of free Pam18, as increased motor activity was slightly less apparent in the *tim17Δ* background compared to the *tim17Δ*/*pam18Δ* double mutant. The fact that constitutive association of Pam18 to Tim17 led to increased motor activity, but at the same time a moderately reduced matrix import efficiency suggested that affecting dynamics of Pam18 is not crucial for regulation of mtHsp70 but rather affects transport in a different manner.

### Translocase association of Pam18 affects lateral sorting

**Fig. 3:**
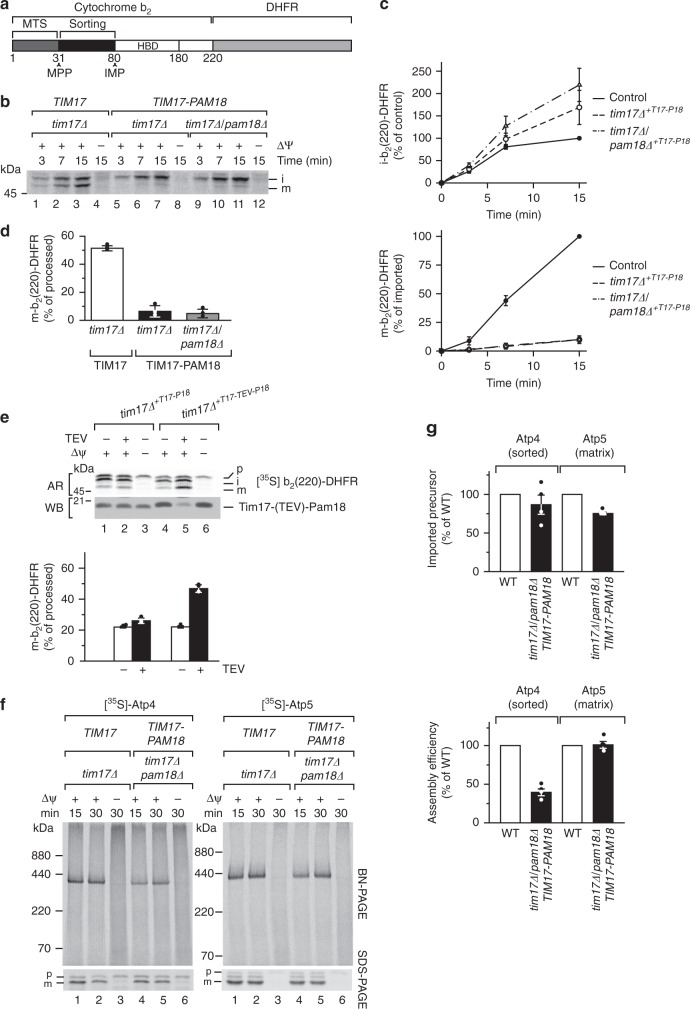
Pam18 affects lateral insertion of membrane-targeted precursors. **a** Schematic presentation of b_2_(220)-DHFR precursor. MTS, mitochondrial targeting signal; MPP, mitochondrial processing peptidase cleavage site; IMP, inner membrane protease cleavage site; HBD, heme-binding domain. **b** [^35^S]-labeled b_2_(220)-DHFR was imported as described in Fig. 2. **c** Quantification of intermediate (upper) and mature (lower) form of b_2_(220)-DHFR from **b**. **d** Quantification of the b_2_(220)-DHFR import after 15 min from **b** (processed = i + m); i, intermediate, m, mature; *tim17∆*^*+T17-P18*^ *tim17∆* + *TIM17–PAM18*. Results are shown as mean ± SEM, *n* = 3. **e** Import of [^35^S]-labeled b_2_(220)-DHFR into Tim17–Pam18 and Tim17-TEV–Pam18 (TEV cleavage site was introduced between Tim17 and Pam18). Mitochondria were swollen and TEV protease treated. Subsequently, precursors were imported for 15 min. Samples were analyzed by SDS-PAGE and digital autoradiography and western blotting. Results are shown as mean ± SEM, *n* = 3. *tim17∆*^*+T17-P18*^ *tim17∆/pam18∆* + *TIM17–PAM18*, AR, autoradiography; WB, western blot. **f** After import of [^35^S]-labeled Atp4 (membrane protein) and Atp5 (soluble matrix protein), samples were analyzed by blue-native PAGE/SDS-PAGE and subjected to digital autoradiography. **g** Quantification from **e**, upper panel, imported precursor after 30 min (SDS-PAGE), lower panel assembly efficiency normalized to import. Results are shown as mean ± SEM, *n* = 4

To investigate the import phenotype, we utilized the sorted inner membrane model protein b_2_(220)-DHFR (Fig. 3a). This protein is processed twice during import. In the matrix, the presequence is cleaved by the matrix processing peptidase (MPP). Subsequently, after the precursor has been inserted into the inner membrane by the TIM23 complex, the intermembrane space protease (IMP) cleaves the precursor a second time^33^. This processing pattern allows us to discriminate and kinetically resolve anterograde precursor movement by N-terminal presequence processing and succeeding lateral membrane insertion indicated by the second processing event. Upon import of the radiolabeled precursor into wild -type mitochondria, both processed forms, referred to as intermediate and mature form, were readily detectable revealing both transport events (Fig. 3b). In *tim17∆* and *tim17∆*/*pam18∆*-mutant mitochondria containing the Tim17–Pam18 fusion, the intermediate form was efficiently generated, demonstrating precursor transport across the inner membrane by the TIM23 complex. Astonishingly, the second processing step was almost completely blocked (Fig. 3b). Quantification of both forms revealed that while the intermediate form accumulated in both mutants, compared to the wild-type, less than 10% of the mature form was generated (Fig. 3c). In line with this, while in the wild type situation roughly 50% of the precursor were processed twice, this was only true for around 5% in the mutant strains (Fig. 3d). To exclude that processing of cytochrome *b*_2_ by IMP was affected due to an indirect effect on the activity of this enzyme, we generated a yeast strain in which a Tobacco Etch Virus (TEV) cleavage site was introduced between Tim17 and Pam18 in the fusion construct. When the outer membrane of mitochondria was ruptured by osmotic swelling, processing to the mature form was improved in the TEV-treated sample, but not in the strain lacking the TEV site (Fig. 3e). Accordingly, the observed strong reduction of the second processing step in the presence of the Tim17–Pam18 fusion was not due to impaired IMP activity. Based on these observations, we concluded that the constitutive association of Pam18 to Tim17 did not affect anterograde protein transport of the polypeptide but rather lateral transport of the precursor into the membrane.

To support this conclusion, we utilized native substrates of the presequence translocase. Atp4 and Atp5 are subunits of the peripheral stalk of the F_1_F_o_-ATP synthase. While Atp4 is an integral membrane protein, Atp5 is a soluble matrix exposed component. In *tim17∆*/*pam18∆*-mutant mitochondria, containing the Tim17–Pam18 fusion, both proteins were imported and processed by MPP with only a slight reduction (Fig. 3f, g). To assess completion of the membrane insertion process, we analyzed protein assembly into the F_1_F_o_-ATP synthase by blue-native PAGE (BN). After 30 min of assembly, the soluble Atp5 integrated into the F_1_F_o_-ATPsynthase with the same efficiency in control and mutant mitochondria (corrected for the mild import defect in the quantification). In contrast, assembly of Atp4 was reduced by 60% in mutant mitochondria. Hence, while the initial translocation across the TIM23 complex was not affected, Atp4 did not assemble efficiently into the ATPase, likely due to the inner membrane sorting defect.

**Fig. 4:**
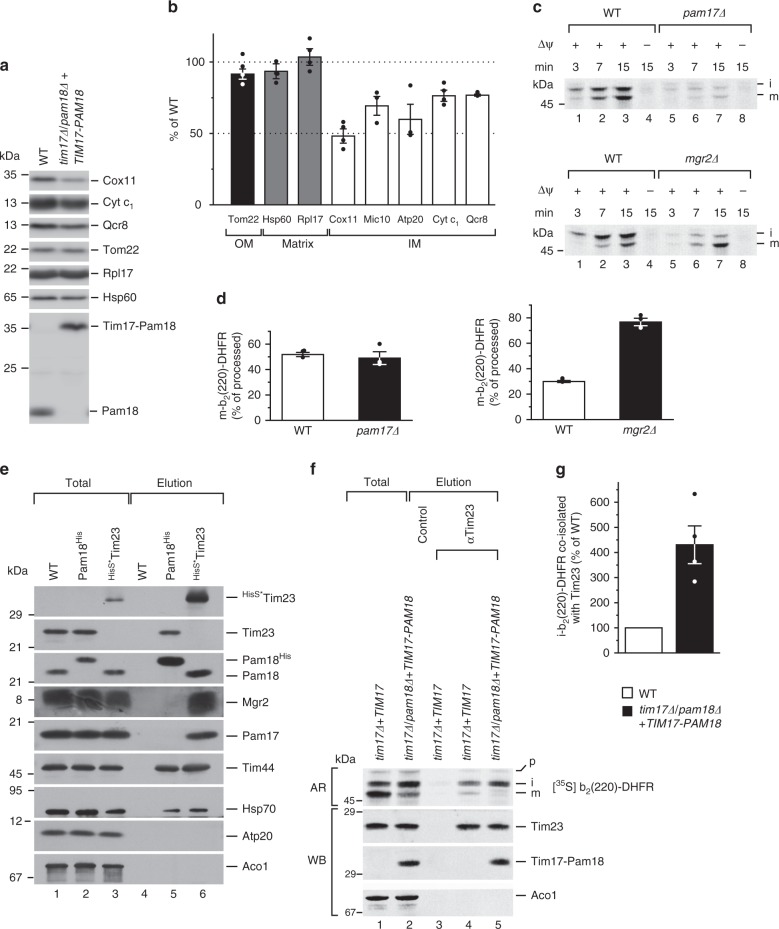
Pam18 regulates the lateral gate. **a** Yeasts were shifted to glycerol medium and grown for 4 h (Mic10 and Atp20) or 6 h (all other tested proteins). Cell lysates were analyzed for levels of indicated outer membrane (OM), matrix or inner membrane (IM) proteins by western blot. **b** Quantification of blots from **a**. Amounts of proteins in percentage of WT control. Results are shown as mean ± SEM, *n* = 3 (Mic10 and Atp20) *n* = 4 for all others. **c**, **d** Import of [^35^S]-labeled b_2_(220)-DHFR in *mgr2∆* and *pam17∆* and corresponding wild-type as described in Fig. 2. **e** WT mitochondria or mitochondria containing Pam18^His^ or ^HisS*^Tim23 were solubilized in digitonin buffer and subjected to Ni-NTA affinity chromatography. Samples were analyzed by SDS-PAGE and western blotting. HisS*, His-SUMOstar. **f** After import of [^35^S]-labeled b_2_(220)-DHFR without proteinase K treatment, mitochondria were solubilized in digitonin buffer and subjected to anti-Tim23 immunoisolation. Samples were analyzed by SDS-PAGE, western blotting, and digital autoradiography. AR, autoradiography; WB, western blot. **g** Quantification of co-isolated i-b_2_(220)-DHFR normalized to isolated Tim23. Results are shown as mean ± SEM, *n* = 4; p, precursor; I, intermediate;m, mature

Next, we addressed whether the observed phenotype in membrane protein insertion in organello was recapitulated in the presence of the Tim17–Pam18 hybrid protein in intact cells. To this end, yeast cells were grown on fermentable medium and shifted to non-fermentable carbon source to stimulate mitochondrial biogenesis. Indeed, we found that membrane proteins of different inner membrane protein complexes were specifically reduced, while both outer membrane and matrix control proteins were unaffected. This finding confirms that Pam18 affects membrane protein insertion in vivo (Fig. 4a, b).

### Translocase association of Pam18 controls lateral transport

Our analyses showed that constitutive association of Pam18 to the translocase did not impact motor function per se. However, lateral transport of the precursor into the lipid bilayer was strongly affected. In addition, presence of Pam18 at the TIM23 complex lead to a loss of Mgr2 and Pam17 from the translocase. While Pam17 supports anterograde transport of ∆ψ-hypersensitive precursors into the matrix but is dispensable for lateral protein transport, Mgr2 has been suggested to provide a quality control on laterally released membrane proteins. To exclude that loss of these components indirectly caused the observed lateral sorting defect, b_2_(220)-DHFR was imported into *mgr2∆* and *pam17∆* mitochondria. In agreement with previous observations, import was strongly reduced for both the intermediate and mature forms in *pam17∆* (Fig. 4c upper, d left). Compared to the phenotype of the Tim17–Pam18 fusion, loss of Mgr2 had the opposite transport effect displaying accelerated generation of mature b_2_(220)-DHFR (Fig. 4c lower, d right and ref. ^12^). Accordingly, neither loss of Pam17 nor Mgr2 explained the reduction of sorting efficiency in the presence of the Tim17–Pam18 fusion.

The loss of Mgr2 from the TIM23 complex when Pam18 was constitutively present suggested that these two proteins bind to the transport channel in a mutually exclusive manner during lateral transport of membrane proteins. To test this, we isolated the TIM23 complex via His-tagged Tim23 (^His^SUMOstar-Tim23) and His-tagged Pam18. In both samples, channel and motor components (Tim44, Hsp70, and Tim23) were efficiently isolated. However, while Tim23 co-purified Mgr2 and Pam17, neither of these could be detected when the translocase was isolated via Pam18. Thus, similar to Pam17, which recently was shown to only bind to Tim23 in the absence of the import motor, Mgr2 is only found translocase associated in the motor-free form (Fig. 4e). Accordingly, Pam18 and Mgr2 define two distinct states of the translocase and potentially associate through overlapping binding sites.

Our data are in agreement with a model in which Pam18 controls the release of membrane proteins through the lateral gate into the lipid bilayer. What happens to sorted precursors that are not released into the inner membrane? Our results can be interpreted in two ways. The precursors could either be mistargeted into the mitochondrial matrix or be trapped in the TIM23 complex. To test this, we imported the inner membrane protein Ina22 into wild-type and Tim17–Pam18 mitochondria, ruptured the outer membrane, and assessed mistargeting to the matrix by protease sensitivity of the substrate. Similar minute amounts of Ina22 were found protected in both strains (Supplementary Fig. 1a). Accordingly, in the presence of Tim17–Pam18, Ina22 was not mistargeted to the mitochondrial matrix, which would lead to an increase in protease protection.

To assess an arrest in the translocase channel, we imported b_2_(220)-DHFR in the presence of MTX into mitochondria containing the constitutively translocase-associated Pam18. Subsequently, we isolated the TIM23 complex. As expected, in the mutant strain four times more of the intermediate form of the precursor was isolated with the translocase compared to the wild-type control (Fig. 4f, g). We conclude that Pam18 prevents the lateral precursor transport from the translocase and thus traps the polypeptide in a translocase-spanning manner.

## Discussion

In order to maintain organelle homeostasis, proteins have to be targeted to and imported into organelles, which includes anterograde translocation across and lateral transport into membranes. In most cases, membrane-embedded protein complexes mediate transport of proteins to their target destinations and, with a few exceptions, these complexes can transport both soluble proteins across or hydrophobic proteins into membranes. Different modes of protein insertion into membranes exist, depending on the nature of the precursor. One of the most common principle is transport through a lateral gate, which involves lateral opening of the import channel toward and concomitant release of the precursor into the lipid bilayer. This has been described extensively for the SecYEG and Sec61 translocon in the bacterial and mammalian ER, respectively, but was also proposed for other systems including the mitochondrial TIM23 complex^34–38^.

The TIM23 complex exists in at least two forms, the TIM23^SORT^ form, which is dedicated to insertion of membrane proteins and the TIM23^MOTOR^ form, which drives full protein translocation at the expense of ATP. It is established that matrix transport of soluble proteins cannot occur in the absence of the import motor, however, if the import motor also affects transport of membrane proteins remained elusive. Here we show that in addition to its function in motor-dependent precursor translocation, the J-protein Pam18 also regulates lateral membrane proteins insertion into the lipid phase. A covalent linkage between Pam18 and the channel component Tim17 delays lateral release of hydrophobic proteins into the mitochondrial inner membrane. Pam18 is the only motor component that contains a membrane-spanning α-helix and is connected to the import channel on both sides of the translocase. This raises the possibility that the transmembrane domain of Pam18 is directly involved in closing the lateral gate when associated with the TIM23 complex. In line with this, Mgr2, which is involved in precursor quality control and affects membrane protein insertion, is displaced from the translocase when Pam18 is covalently fused to the channel component Tim17. This finding suggests overlapping binding sites of these proteins at the lateral gate. Interestingly, Mgr2 was not co-purified with Pam18^His^, indicating that both proteins define different states of the translocase.

We propose that during transport of matrix-targeted proteins, recruitment of Pam18 blocks the lateral gate thereby promoting anterograde transport of the precursors into the mitochondrial matrix. Once a hydrophobic transmembrane span gets arrested in the TIM23 channel, Pam18 dissociates allowing Mgr2 recruitment, which mediates quality control during membrane protein insertion. This would also explain why in *mgr2∆* mitochondria, the dynamic recruitment of Pam18 is reduced during precursor translocation. This is especially interesting for membrane proteins that have N-terminal matrix domains which have to be imported by the motor complex but are lateral released into the inner membrane such as Cox5a in yeast. For such precursors, the TIM23 complex has to identify the transmembrane segment, which leads to a transfer arrest, probably associated with motor dissociation and Mgr2 association. Strikingly, the transmembrane segment of Pam18 has a cluster of only mildly hydrophobic residues, which could be exposed into the water-filled transport channel when Pam18 is TIM23 associated. Our findings explain a surprising previous observation. The deletion of the IMS domain of Pam18 was found to increase import of membrane proteins^39^. Since the IMS domain of Pam18 couples the protein to Tim17, abrogating this interactions site affects association of Pam18 with the import channel and concomitantly allows for a more efficient lateral transport.

Interestingly, despite the clear import defect for membrane proteins in vivo after switching yeast to non-fermentable carbon sources, we did not observe a decrease in membrane potential in isolated mitochondria (Fig. 2a). On the one hand, this could be due to the fact that the constitutive presence of Pam18 at the lateral gate does not completely block lateral sorting (Fig. 4a, b). On the other hand, a recent study showed that in response to accumulation of membrane-targeted TIM23 substrates, yeast cells mount a transcriptional response, which ultimately helps to remove stalled precursors and thereby improve import efficiency^40^. It would be interesting to address if this process also supports the transport process in the Tim17–Pam18 mutant.

To what extent the dynamics of Pam18 (and Pam16) is important for protein translocation is not known. In the Tim17–Pam18 fusion construct, Pam18 cannot be exchanged at the translocase, even if endogenous Pam18 is still present. Nevertheless, yeast cells are viable and only display a mild growth defect while transport of soluble proteins is reduced by roughly 50%, which might be attributed to reduced motor dynamics. Interestingly, when motor-depended backsliding of a stalled precursor was assessed in this strain, mitochondria displayed an increase in protease-protected precursor, which points toward increased motor function. Since this in contrast to the import phenotype for matrix proteins, we suggest that in the Tim17–Pam18 strain reduced motor dynamics lead to a prolonged stimulation of Hsp70, which is bound to both precursor and translocase. As a result, we observe a decrease in anterograde movement of the precursor while backsliding is prevented.

**Fig. 5:**
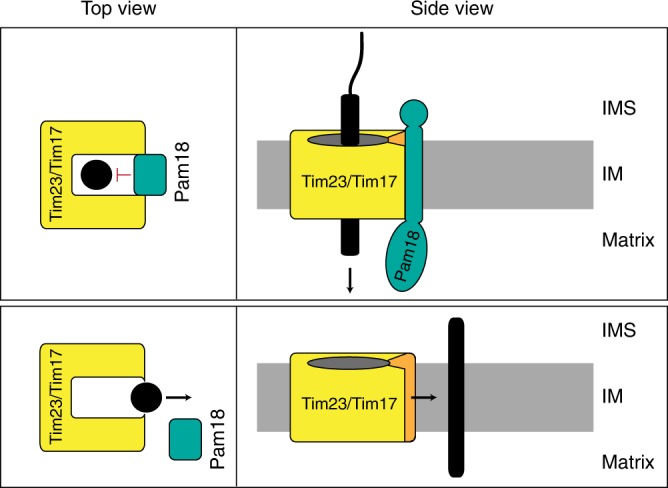
Pam18 controls the lateral gate promoting anterograde protein transport. Model of how Pam18 might affect release of membrane-targeted proteins into the lipid bilayer. When Pam18 is bound together with other motor components, the lateral gate is closed, which promotes matrix translocation of substrates. IMS, intermembrane space; IM, inner membrane

In conclusion, for translocation of precursors across or into the inner mitochondrial membrane, opening of the lateral gate is tightly regulated by the import motor constituent Pam18. Hence, motor recruitment switches the direction of precursor movement not only by providing energy for anterograde transport, but also by restricting the access to the lateral gate (Fig. 5). It will be important to define if a similar regulation of lateral precursor movement by associated transmembrane segments exists in chloroplast and the endoplasmic reticulum. Interestingly, in both cases, for the mammalian Sec complex and the TIC complex, membrane-bound J-proteins have been reported.

## Methods

### Yeast strains and handling

The wild-type yeast strain YPH499 (*MAT*a, *ade2-101*, *his3-∆200*, *leu2-∆1*, *ura3-52*, *trp1-∆63*, and *lys2-801*) (ATCC® 204679™) and mutants derived from it were used in this study. *mgr2∆*, *pam17∆*, and Pam18^His^ have been described previously^21,31,41^. Yeast strains were grown in YP medium (1% yeast extract, 2% peptone) containing 2% glucose (YPD) or 3% glycerol (YPG) at 30 °C. *mgr2∆* and corresponding WT were grown at 25 °C. For generation of His-SUMOstar-Tim23 (^HisS*^Tim23), a His-tag (MTSKHHHHSGHHHTGHHHHSGSHHHGS) together with SUMOstar^42^ was fused to the *TIM23* gene. This construct together with 511 bp upstream and 308 bp downstream of the open reading frame were cloned into pRS413. For generation of the Tim17–Pam18 strain and modifications thereof, *TIM17* + 996 bp upstream were cloned with *Eco*RI and *Bam*HI into pFL39. In a second round, *PAM18* + 1000 bp downstream were cloned with *Bam*HI and *Sal*I into the vector. The resulting plasmid (pCS164) and wild-type *TIM17* containing plasmids were transformed into YPH499 in which *tim17* deletion was rescued with wild-type *TIM17* expressed from *URA3*-containing plasmid. The *URA3-*containing plasmid was shuffled out by selecting yeast on 5-FOA-containing medium. In a second round, *PAM18* was deleted using pFA6a HIS and *PAM16* with pFA6a hphNT1. For generation of TEV cleavable Tim17–Pam18, the TEV site (ENLYFYG) was introduced by overlap PCR downstream of the *Bam*HI site. Primers used in this study are listed in the Supplementary Table 1.

For preparation of whole cell lysates, yeast cells were grown in YPD medium at 30 °C over night, 5 OD_600_ harvested the next day, cells sedimented, washed with water, and suspended in 300 µl H_2_O. After addition of 100 µl 50% TCA, cells were incubated for 15 min on ice, precipitated proteins pelleted, washed with 80% ice-cold acetone, pelleted again and resuspended in buffer containing 1% SDS, 100 mM NaOH. For analysis of in vivo import defects, cells were grown over night in YPD medium at 30 °C. Next morning cells were diluted to OD_600_ = 0.3 in YPG medium, grown for 4–6 h and analyzed as described before.

### Import of precursor proteins

Precursor proteins were radiolabeled by translation in the presence of [^35^S]-methionine using rabbit reticulocyte lysate (Promega). Isolated mitochondria were mixed in import buffer (250 mM sucrose, 10 mM MOPS/KOH pH 7.2, 80 mM KCl, 2 mM KH_2_PO_4_, 5 mM MgCl_2_, 5 mM methionine, and 3% fatty acid-free BSA) with 2 mM ATP and 2 mM NADH. Aliquots of 1 µM valinomycin, 8 µM antimycin A, and 20 µM oligomycin were used to dissipate the membrane potential to stop import reactions, after which Proteinase K (PK, 20 µg/ml) treatment was performed to remove, not imported proteins. After 10 min incubation on ice, 2 mM phenylmethylsuophonyl fluoride (PMSF) was added for 10 min on ice to inactivate PK. After pelleting mitochondria and washing with SEM (250 mM sucrose, 20 mM MOPS pH 7.2, and 1 mM EDTA), import was analyzed by SDS-PAGE or BN-PAGE (for BN samples, PK treatment was omitted) and autoradiography. Quantifications were performed using ImageQuant TL (GE Healthcare) using a rolling ball background subtraction.

For import after TEV cleavage, mitochondria were resuspended in import buffer lacking sucrose and incubated with 60 µg/ml TEV protease and kept on ice for 30 min. Subsequently, 2 mM NADH and ATP were added following the import reaction.

For swelling experiments, precursor was imported for 15 min, import stopped with AVO and samples split into two equal samples. After SEM wash, samples were treated with EM buffer (20 mm MPOS pH 7.2, and 1 mM EDTA) for 20 min on ice and 5 µg PK was added to 100 µl EM buffer. After 15 min incubation, 2 mM PMSF was added for 15 min, mitochondria were washed with SEM and analyzed by SDS-PAGE and autoradiography.

### Membrane potential measurements

Mitochondrial membrane potential was assessed using the potential sensitive dye 3,3′-dipropylthiadicarbocyanine iodide (DiSC_3_(5)). For this, mitochondria were diluted in buffer containing 600 mM sorbitol, 1% (wt/vol) BSA, 10 mM MgCl_2_, and 20 mM KPi, pH 7.4 to a concentration of 166 µg/ml. Changes in fluorescence were recorded using a F-7000 fluorescence spectrophotometer (Hitachi) at 25 °C with excitation at 622 nm, emission at 670 nm, and slits of 5 nm. To 500 µl of buffer in a cuvette with DiSC_3_(5), 83 µg of mitochondria were added first and, after fluorescence signal reached a stable signal, 1 µM valinomycin was added. The difference in fluorescence before and after the addition of valinomycin was used to compare relative membrane potential between strains.

### Import-driving activity

Import-driving activity was assessed as previously described^43^. In brief, mitochondria were mixed in import buffer supplemented with 5 mM creatine phosphate (CP) and 0.01 mg/ml creatine kinase (CK). Radiolabeled b_2_(167)_∆_-DHFR was imported at 25 °C for 15 min in the presence of 5 µM methotrexate (MTX) and membrane potential was dissipated using 1 µM valinomycin. A sample (∆*t* = 0) was taken prior to chase for 1, 5, and 10 min and PK was added for 15 min on ice. After protease inhibition with PMSF, samples were analyzed by SDS-PAGE and autoradiography. The amount of PK-resistant intermediate was quantified and normalized to the amount of generated intermediate (∆*t* = 0) for each strain.

### Protein complex isolation

Isolation of TIM23 complex using Tim23-specific serum was carried out essentially as described^44^. For this, mitochondria were resuspended to 1 mg/ml in solubilization buffer (20 mM Tris/HCl pH 7.4, 150 mM NaCl, 10% glycerol (w/v), 1 mM PMSF, and 1% digitonin) and solubilized for 30 min on ice. The soluble fraction was loaded on Protein A sepharose beads cross-linked to Tim23 antibodies, incubated for 30 min to 1 h on a spinning wheel at 4 °C, washed five times (solubilization buffer with 0.3% digitonin) and eluted with double bed volume of 0.1 M glycine pH 2.8. For isolation by His-tags, mitochondria were solubilized as described earlier with 10 mM imidazole, supernatant was loaded on Ni-NTA beads, washed with washing buffer containing 10 mM imidazole and eluted with washing buffer containing 200 mM imidazole.

For TIM23 isolation after import, b_2_(220)-DHFR was imported in the presence of 5 mM methotrexate (MTX) and mitochondria solubilized after an SEM wash without addition of antimycin A, Valinomycin, oligomycin, or Proteinase K.

### Isolation of mitochondria

Mitochondria were isolated as previously described^45^. Yeast were grown in YPG medium at 30 °C and harvested at OD_600_ = 2–3 if not stated otherwise. Pelleted cells were resuspended in buffer A (10 mM DTT, 100 mM Tris/H_2_SO_4_ pH 9.4) and incubated for 30 min at 30 °C. After a washing step, cell wall removed in zymolyase buffer (20 mM KPO_4_ pH 7.4, 1.2 M sorbitol, and 0.57 mg/l zymolyase) for 1–2 h at 30 °C and opened in ice-cold homogenization buffer (600 mM sorbitol, 10 mM Tris/HCl pH 7.4, 1 g/l BSA, 1 mM PMSF, and 1 mM EDTA) using a cell homogenizer. Mitochondrial fraction was obtained by differential centrifugation, resuspended in SEM buffer and frozen in liquid nitrogen.

## Electronic supplementary material

Supplementary Information

## Data Availability

Data supporting the findings of this manuscript are available from the corresponding author on reasonable request.
