# Supplementary material for: Motor recruitment to the TIM23 channel’s lateral gate restricts polypeptide release into the inner membrane
Source: Nat Commun. 2018 Oct 2;9:4028. doi: 10.1038/s41467-018-06492-8 (PMC6168564; doi:10.1038/s41467-018-06492-8)

Supplementary Information

Motor-recruitment to the TIM23 channel's lateral gate restricts polypeptide release into the mitochondrial inner membrane

Alexander Benjamin Schendzielorz[1,2], Piotr Bagosewski[3], Nataliia Naumenko[1,2], Ridhima Gomkale[1,2], Christian Schulz[1,2], Bernard Guiard[4], , Agnieszka Chacinska[3] & Peter Rehling[1,2,5,*]

a

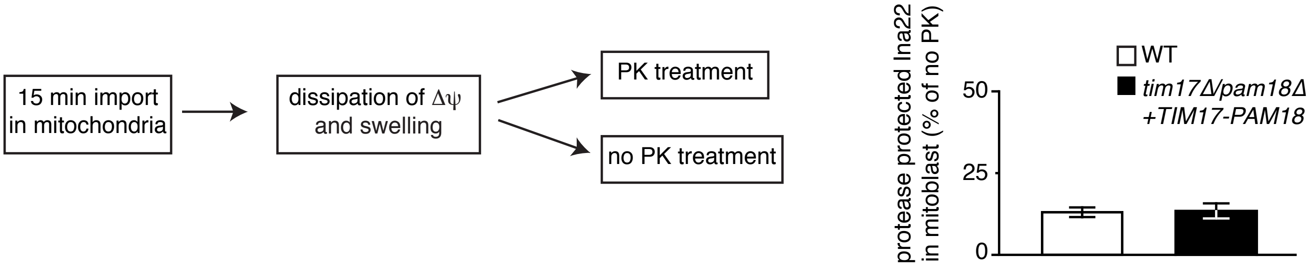

Supplementary Figure 1: Block of lateral gate does not induce missorting of precursor proteins.

a [^{35}S]-labelled Ina22 was imported in mitochondria as described in Figure 2. After stop of import by dissipation of $\Delta\Psi$, mitochondria were washed with SEM and the outer membrane was ruptured by resuspension in hypotonic buffer (EM buffer, 20 mM MOPS pH 7.2, 1 mM EDTA). Samples were split in two and proteinase K was added to one sample. Quantification shows PK protected Ina22 (% of WT). Results are shown as mean ± s.d., n=3

Supplementary Table 1:
List of primers used in this study

Primer name	Sequence	Purpose
priAS213	TCGACGGTATCGATAAGCTTCGATTCCCCACCAAGTATAAGTGT	Gibson cloning of Tim23 + 511 bp 5´ in pRS413 fwd
priAS214	GGCTGCAGGAATTCGATATCAATACCCGAGAGTGAGCGGTTTGT	Gibson cloning of Tim23 + 308 bp 3´in pRS413 rev
priAS215	ACCGCTCACTCTCGGGTATTGATATCGAATTCCTGCAGCCCGGG	Gibson cloning pRS413 vector PCR for Tim23 fwd
priAS216	TTATACTTGGTGGGGAATCGAAGCTTATCGATACCGTCGACCTC	Gibson cloning pRS413 vector PCR for Tim23 rev
priAS358	ATATGGTACCGCCTTATATTCAGGTAAATCACCA	Tim17 + 996 bp fwd primer for TIM17-TEV-PAM18 cloning
priAS359	GCCCTGAAAATACAGGTTTTCGGATCCAGCTTGCAGAGG	Tim17 rev + TEV for overlap PCR of Tim17-TEV-PAM18 construct rev
priAS360	CCGAAAACCTGTATTTTCAGGGCATGAGTTCTCAAAGTAATACTGGT	TEV+Pam18 fwd for overlap PCR of TIM17-TEV-PAM18 construct fwd
CS307	ATATGAATTCGCCTTATATTCAGGTAAATCAC	Cloning of Tim17 in pFL39 fwd
CS308	ATAT GGATCC AGCTTGCAGAGGTTGAGAGGAAGG	Cloning of Tim17 in pFL39 rev
CS309	ATAT GGATCC **ATG**AGTTCTCAAAGTAATACTGGTAATTC	Cloning of Pam18 in pFL39 fwd
CS-86	ATATGTCGACCAAGCTAAATGATTATATAAACGCTA	Cloning of Pam18in pFL39 rev (also rev for TIM17-TEV-PAM18)
CS185-Pam16-S1	AGCTTCGTGACTTGAGCATAAGAAAAACATAGACTTGTTGGATAT AAAATATGCGTACGCTGCAGGTCGAC	Deletion of Pam16
CS186-Pam16-S2	GCATGCTTTCGATAACACTTGTGACGTAATGATGGAGGCTTCCTTG ACTAATCGATGAATTCGAGCTCG	Deletion of Pam16
Pam18-ura-del-fwd	CACAGTTTAATAAGGTTGCATAAACACTTCCACCCGCACAATATCC AGCTGAAGCTTCGTACGC	Deletion of Pam18
Pam18-ura-del-rev	CATATATGCAATTGCAATAACTCATTTTAGGTTCCCGTTTTACCTTA GCATAGGCCACTAGTGGATCTG	Deletion of Pam18
priAS288	ATGTCGTGGCTTTTTGGAGATAAGAC	Tim23 vector PCR for His-SUMOStar gibson fwd
priAS289	GATTGTGTGTGATCTGTTAAACAAGTATAC	Tim23 vector PCR for His-SUMO* gibson rev
priAS292	CTTGTTTAACAGATCACACACAATCATGACTAGCAAGCATCACCATC	PCR for Gibson cloning His-SUMOstar for Tim23 in pRS413 fwd
priAS294	CTTATCTCCAAAAAGCCACGACATGCCACCAATCTGTTCTCTGTG	PCR for Gibson cloning His-SUMOStar for Tim23 in pRS413 rev

For generation of His-SUMOstar-Tim23 (^{HisS*}Tim23), a His-tag (MTSKHHHHSGHHHTGHHHHSGSHHHGS) together with SUMOstar [42] was fused to the *TIM23* gene. For this TIM23 with 511 bp upstream and 308 bp downstream of the open reading frame was cloned into pRS413 by Gibson assembly (priAS213+priAS214 for Tim23, priAS215+priAS216 for vector). In a second round, His-SUMOstar was Gibson cloned at the

5´end of TIM23 (priAS292+priS294 for His-SUMOstar, priAS288+priAS289 for vector). For generation of the Tim17-Pam18 strain and modifications thereof, *TIM17* + 996 bp upstream were cloned with EcoRI and BamHI into pFL39 (CS307+CS308). In a second round, *PAM18* + 1000 bp downstream were cloned with BamHI and SalI into the vector (CS309+CS86). The resulting plasmid (pCS164) and wild type *TIM17* containing plasmids were transformed into YPH499 in which *tim17* deletion was rescued with wild type *TIM17* expressed from *URA3* containing plasmid. The *URA3* containing plasmid was shuffled out by selecting yeast on 5-FOA containing medium. In a second round, *PAM18* was deleted using pFA6a HIS (Pam18-ura-del-fwd + Pam18-ura-del-rev) and *PAM16* with pFA6a hphNT1 (pCS185+pCS186). For generation of TEV cleavable Tim17-Pam18, the TEV site (ENLYFYG) was introduced by overlap PCR downstream of the BamHI site. The resulting PCR product was cloned with KpnI and SalI into pFL39 (priAS358-priAS359-priAS360-pCS86).

Supplementary Figure 2
Original scans of key Western blots and gels presented in the paper

Figure 1c

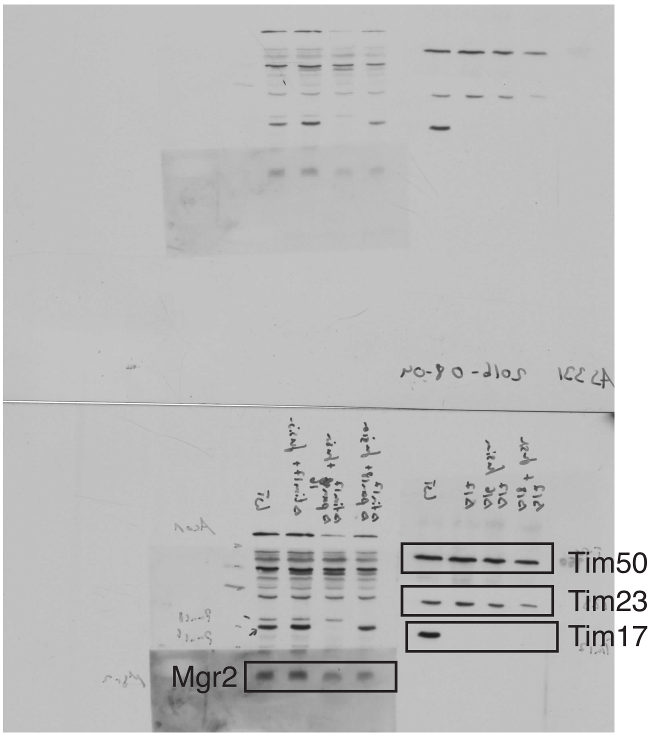

Figure 1c

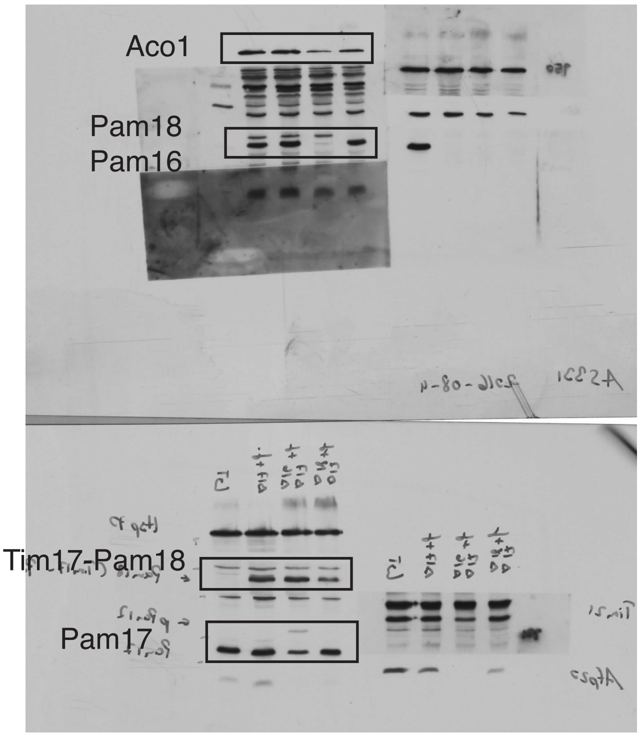

Figure 1d

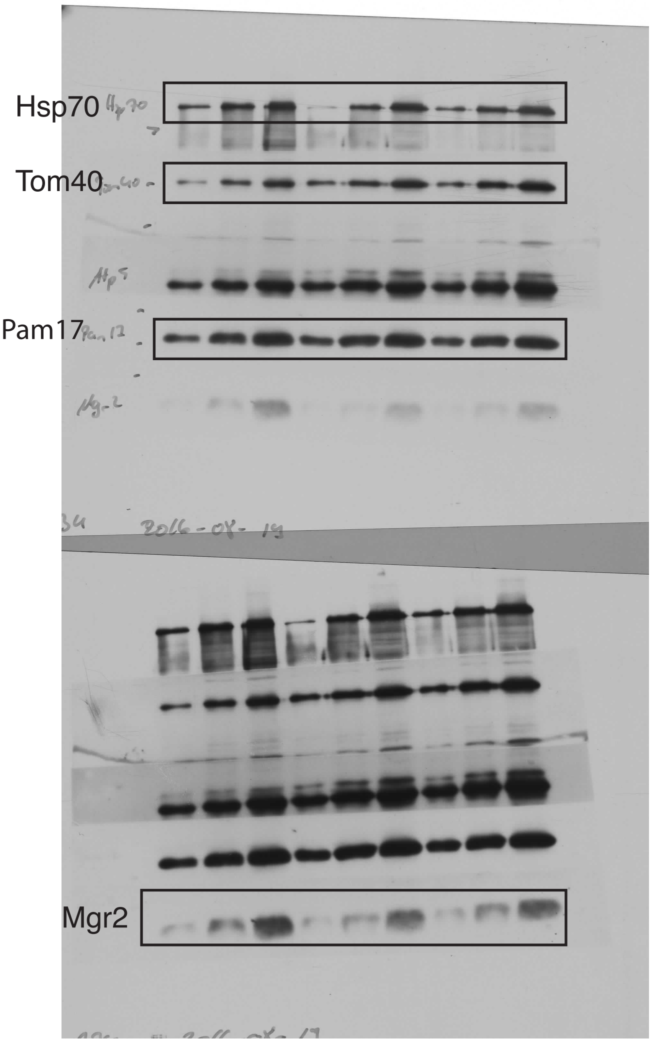

Figure 1d

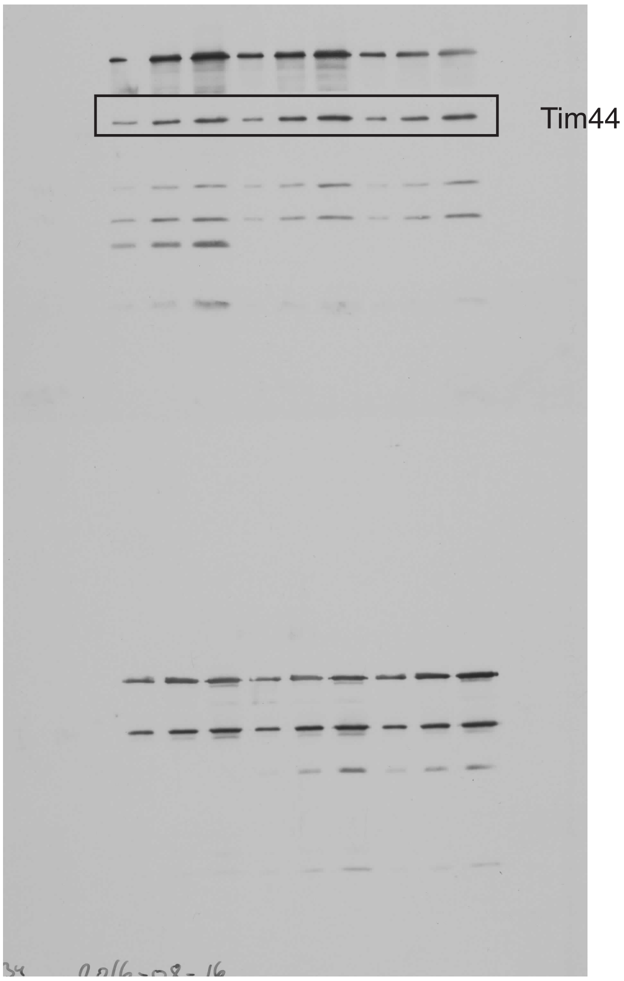

Supplementary Figure 3
Original scans of key Western blots and gels presented in the paper

Figure 1d

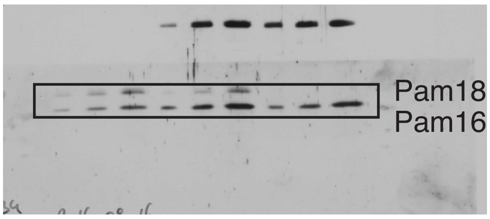

Figure 1d

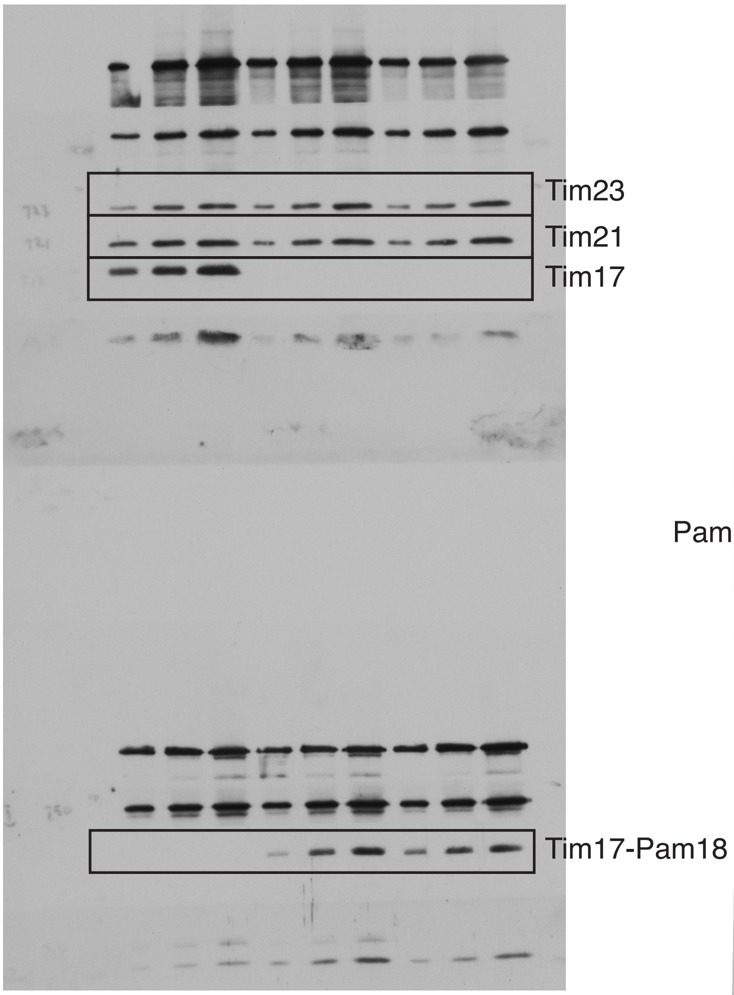

Figure 1e

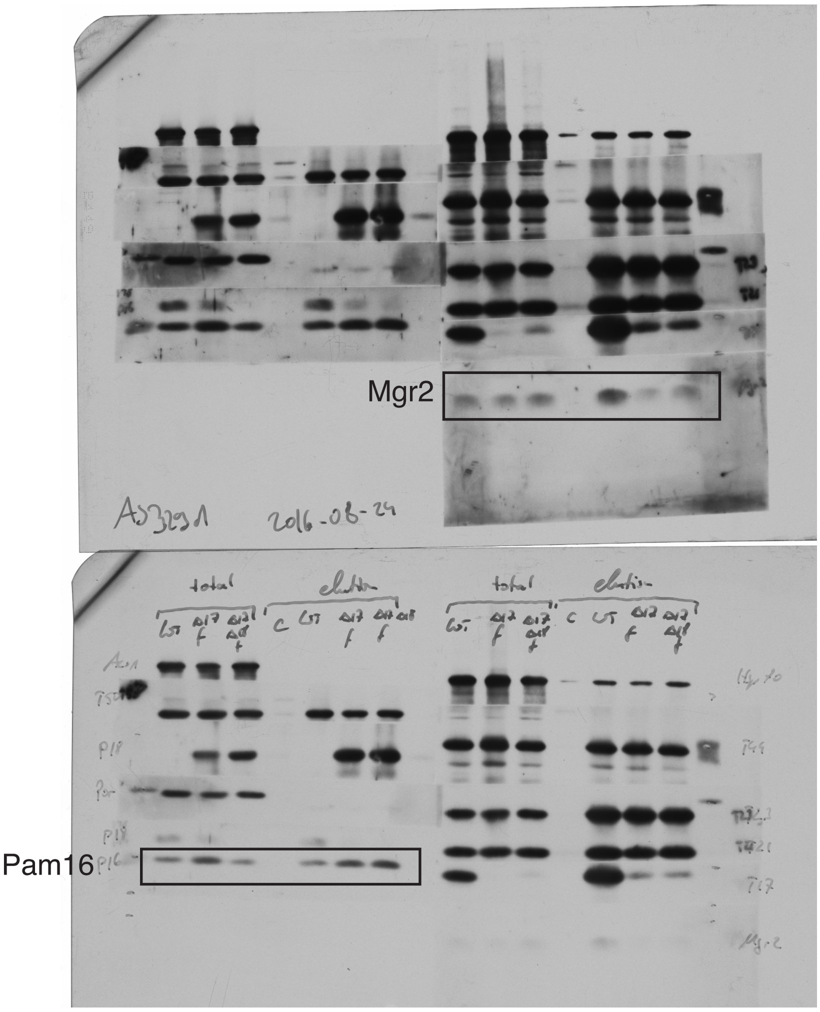

Figure 1e

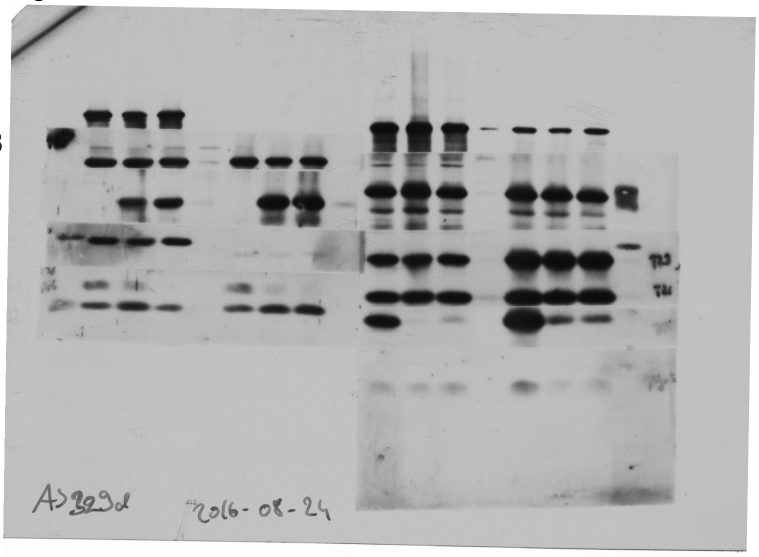

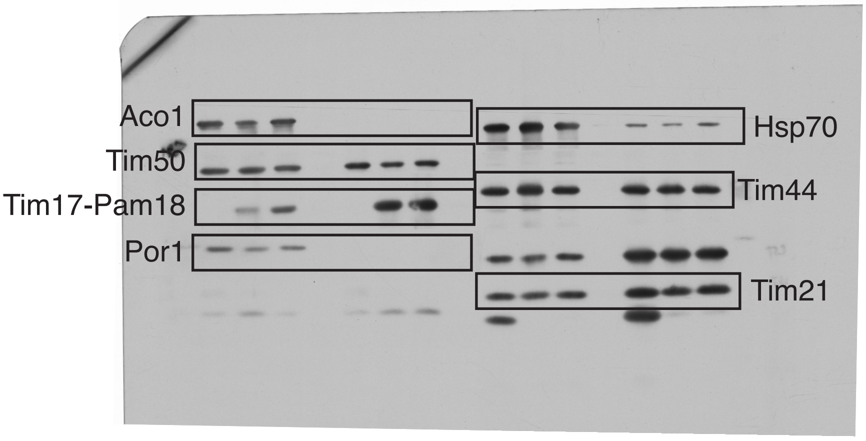

Supplementary Figure 4
Original scans of key Western blots and gels presented in the paper

Figure 1e

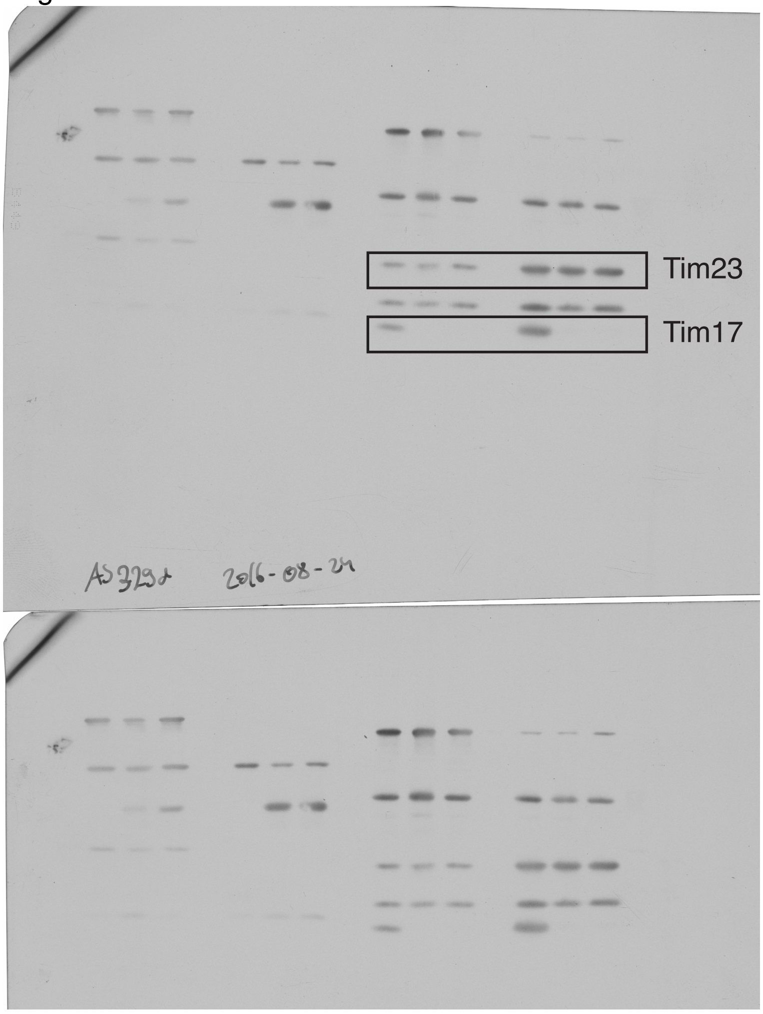

Figure 1e

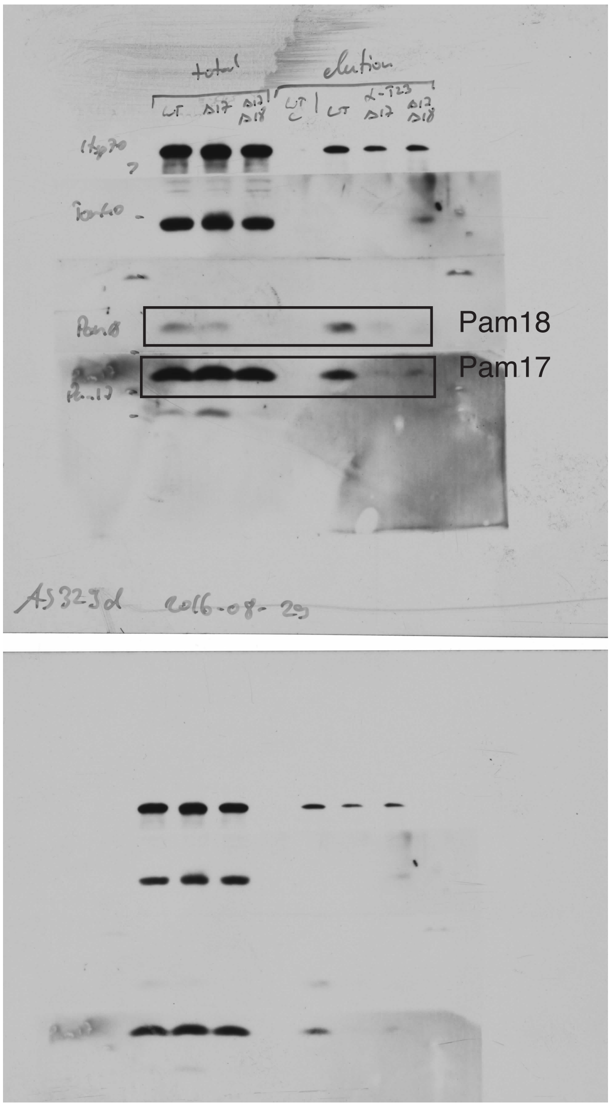

Figure 1e

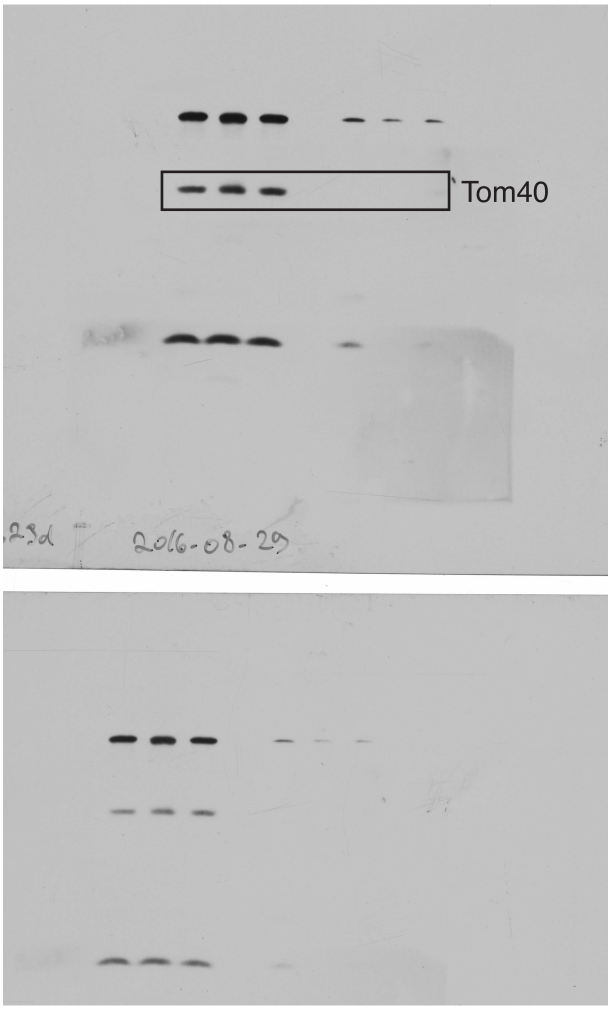

Supplementary Figure 5
Original scans of key Western blots and gels presented in the paper

Figure 2b

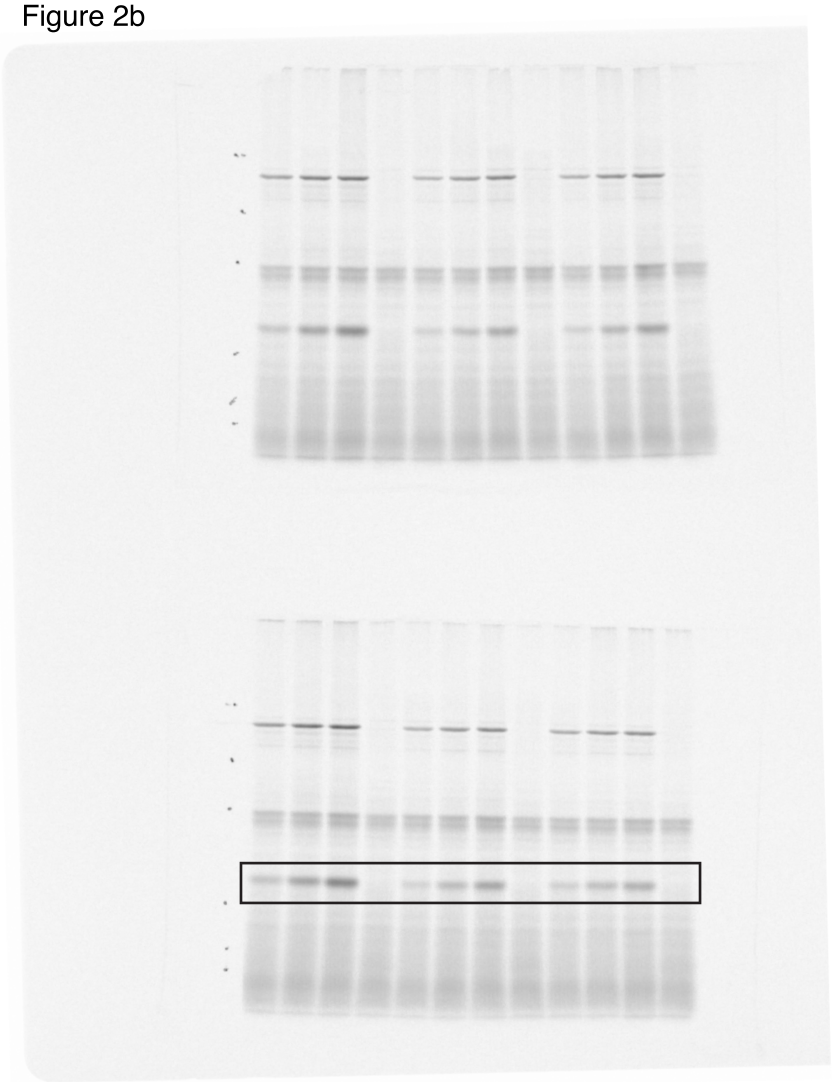

Figure 2c

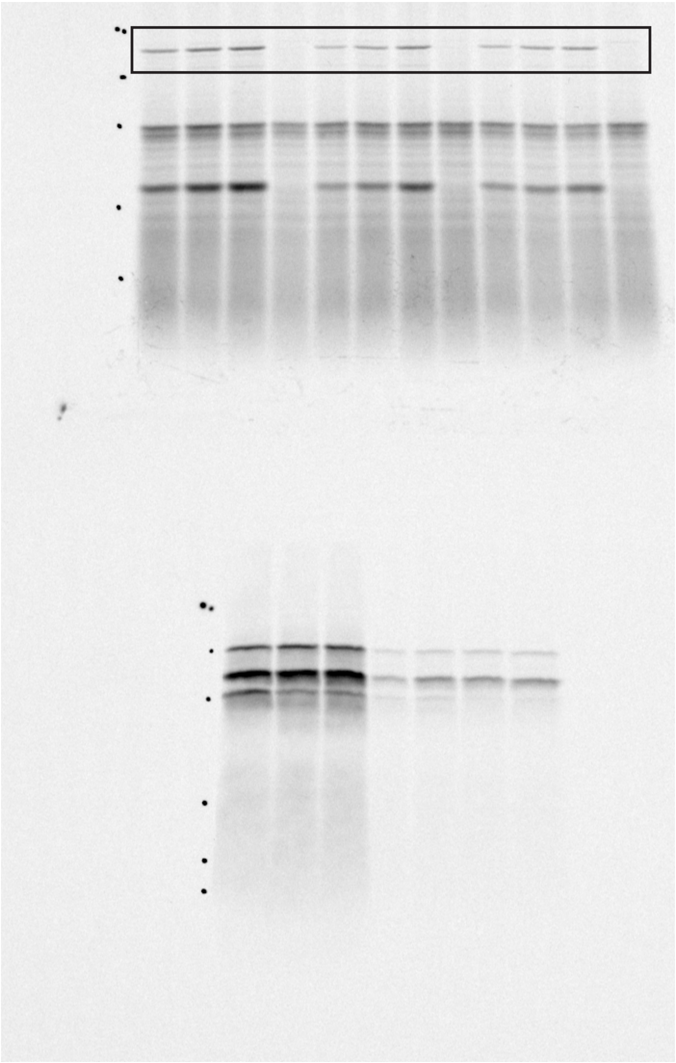

Figure 2d

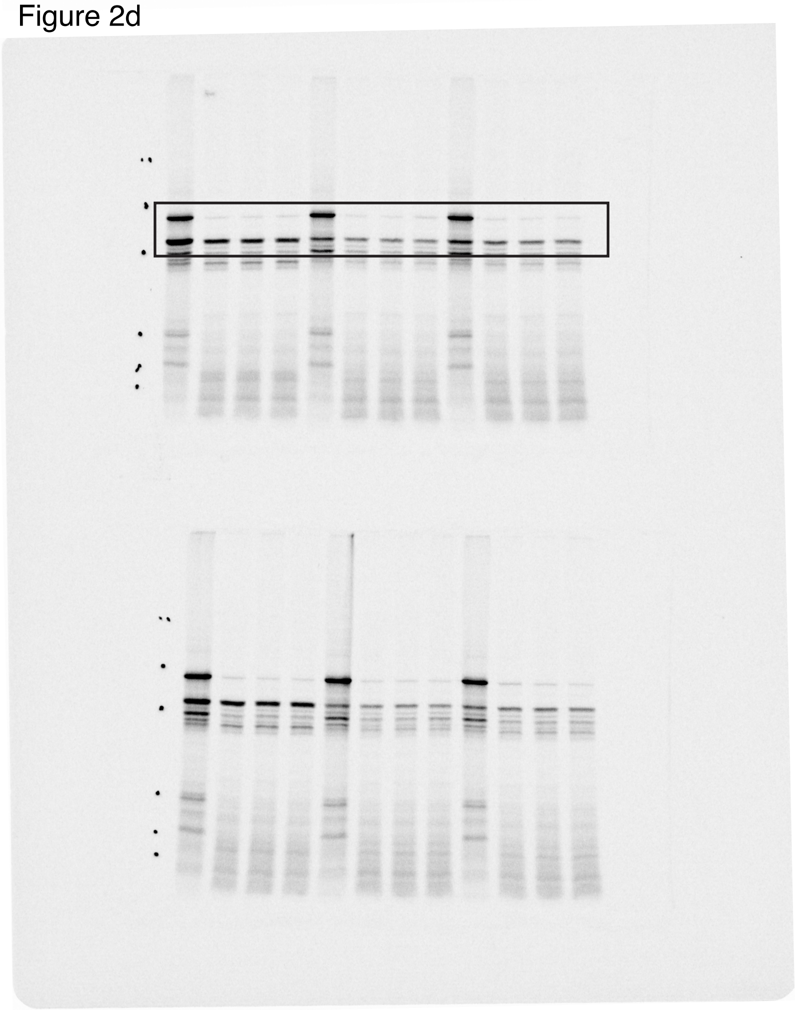

Supplementary Figure 6
Original scans of key Western blots and gels presented in the paper

Figure 3c

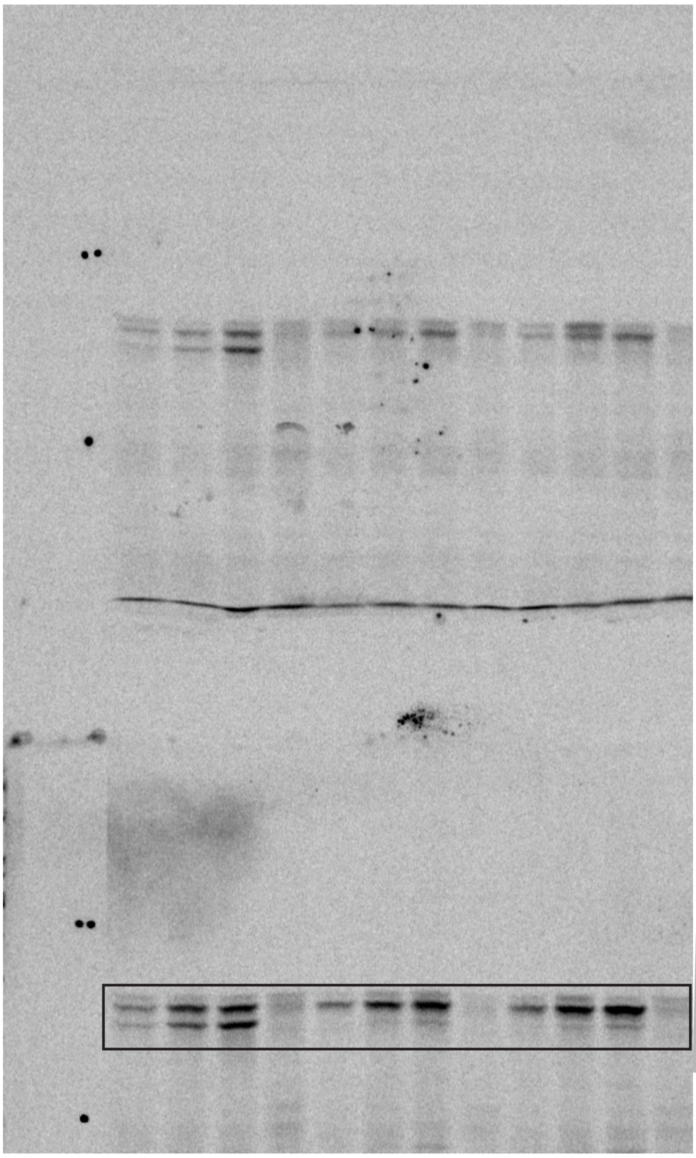

Figure 3e

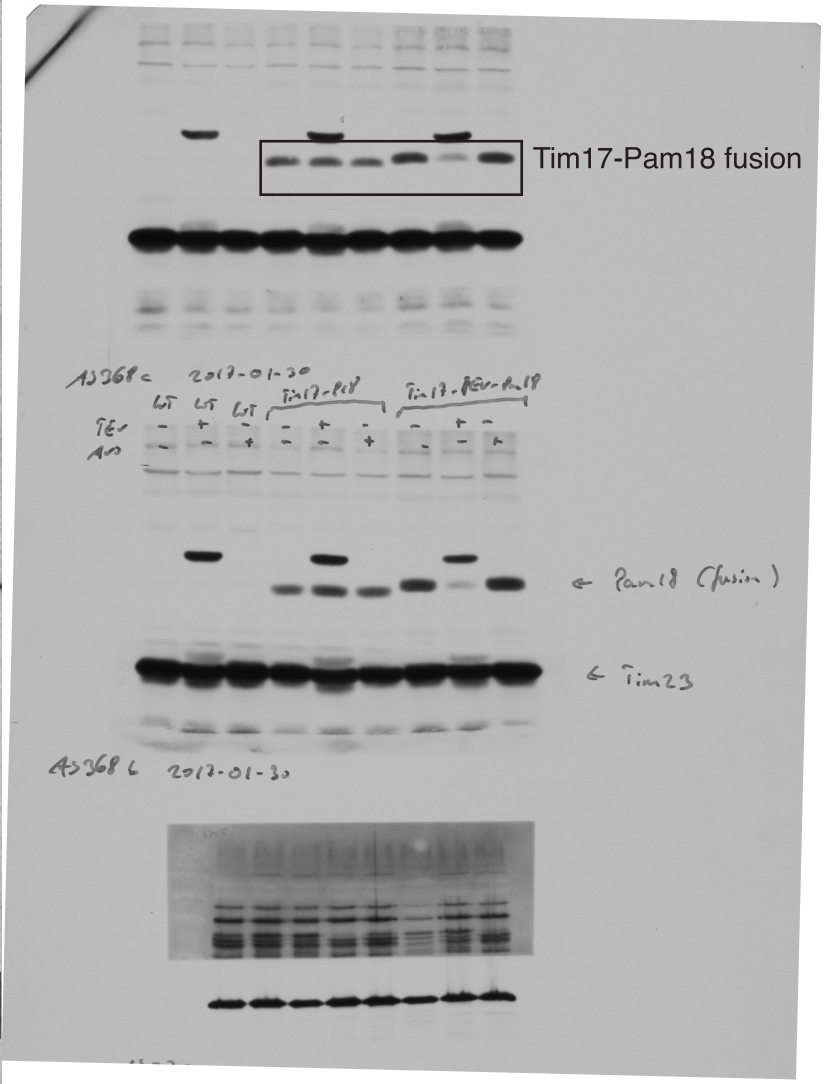

Figure 3e

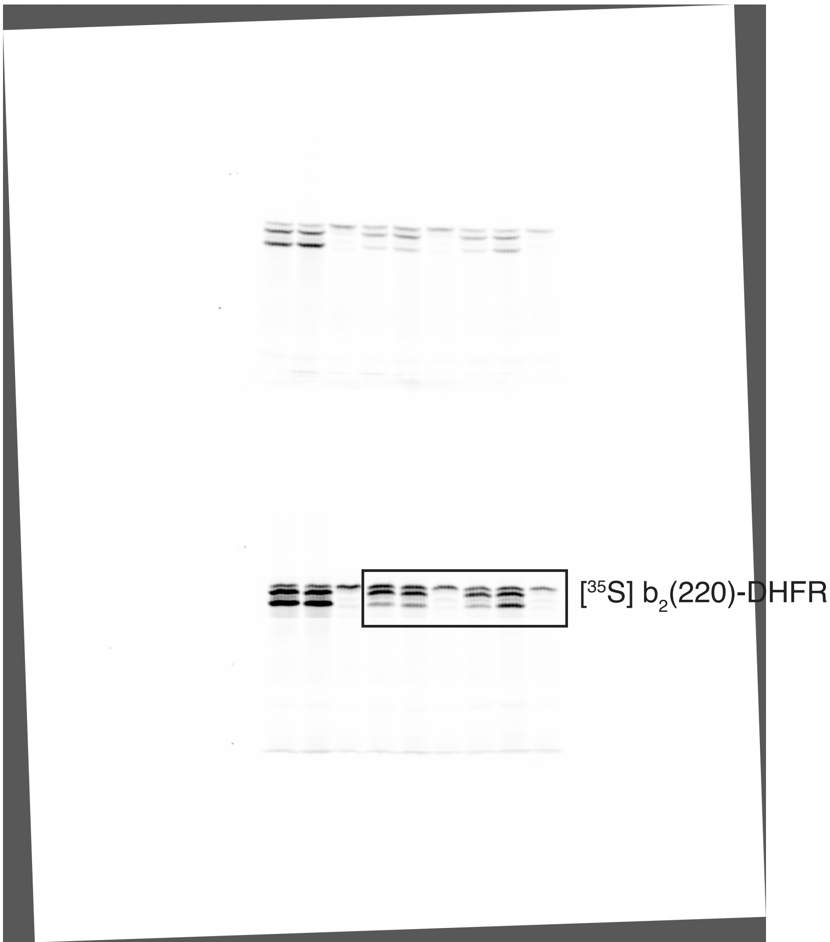

Supplementary Figure 7
Original scans of key Western blots and gels presented in the paper

Figure 3f

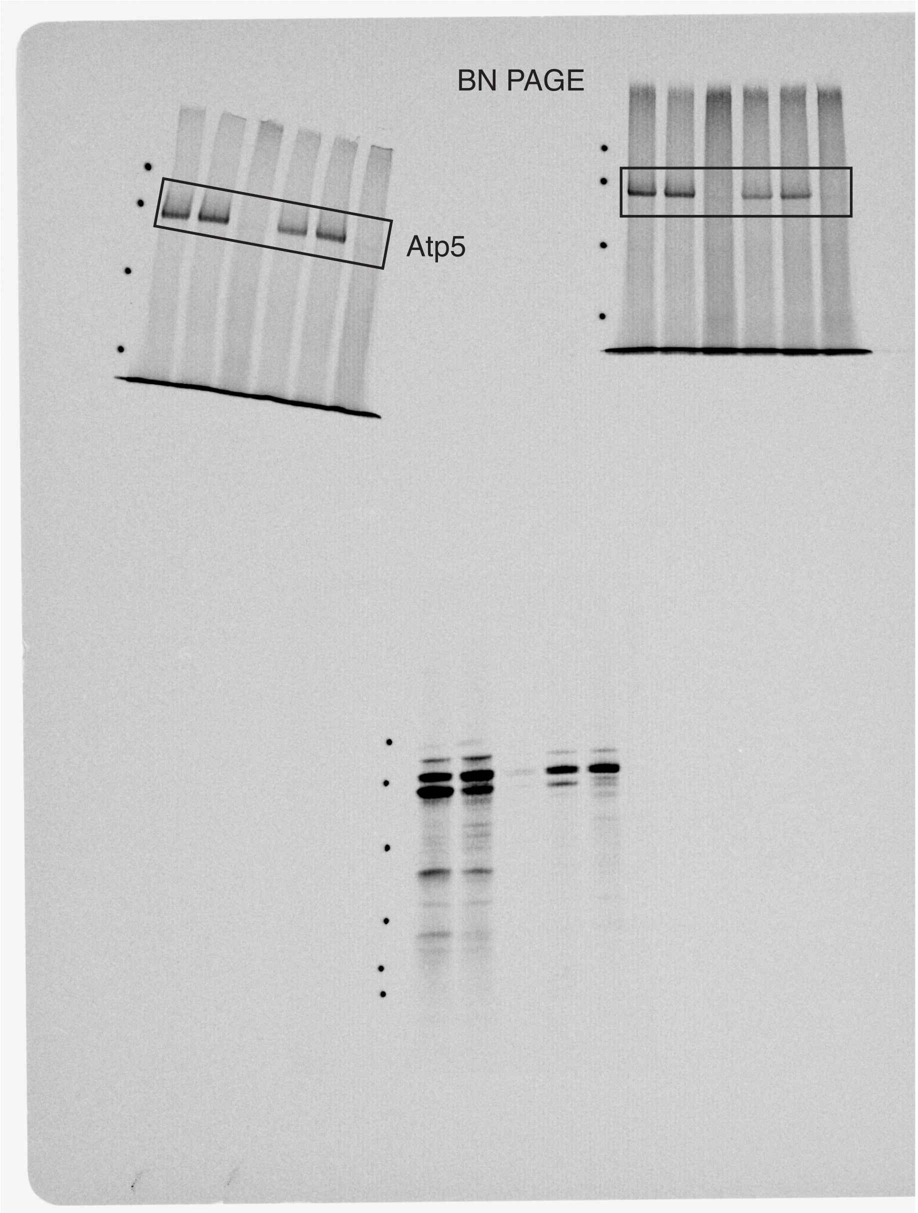

Figure 3f

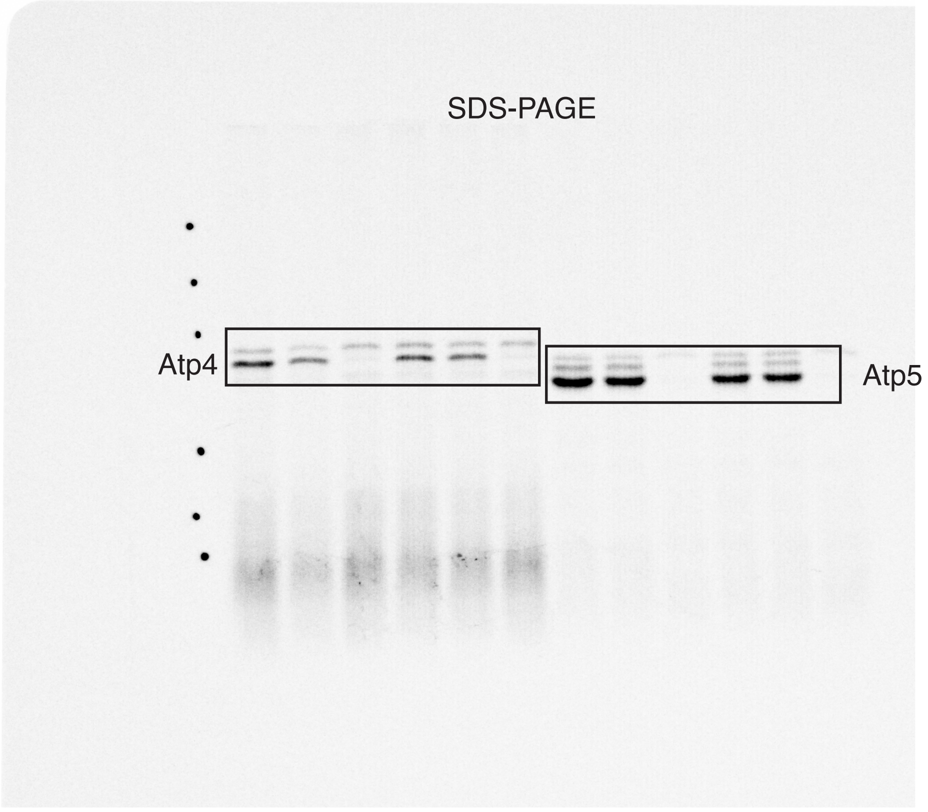

Supplementary Figure 8
Original scans of key Western blots and gels presented in the paper

Figure 4a

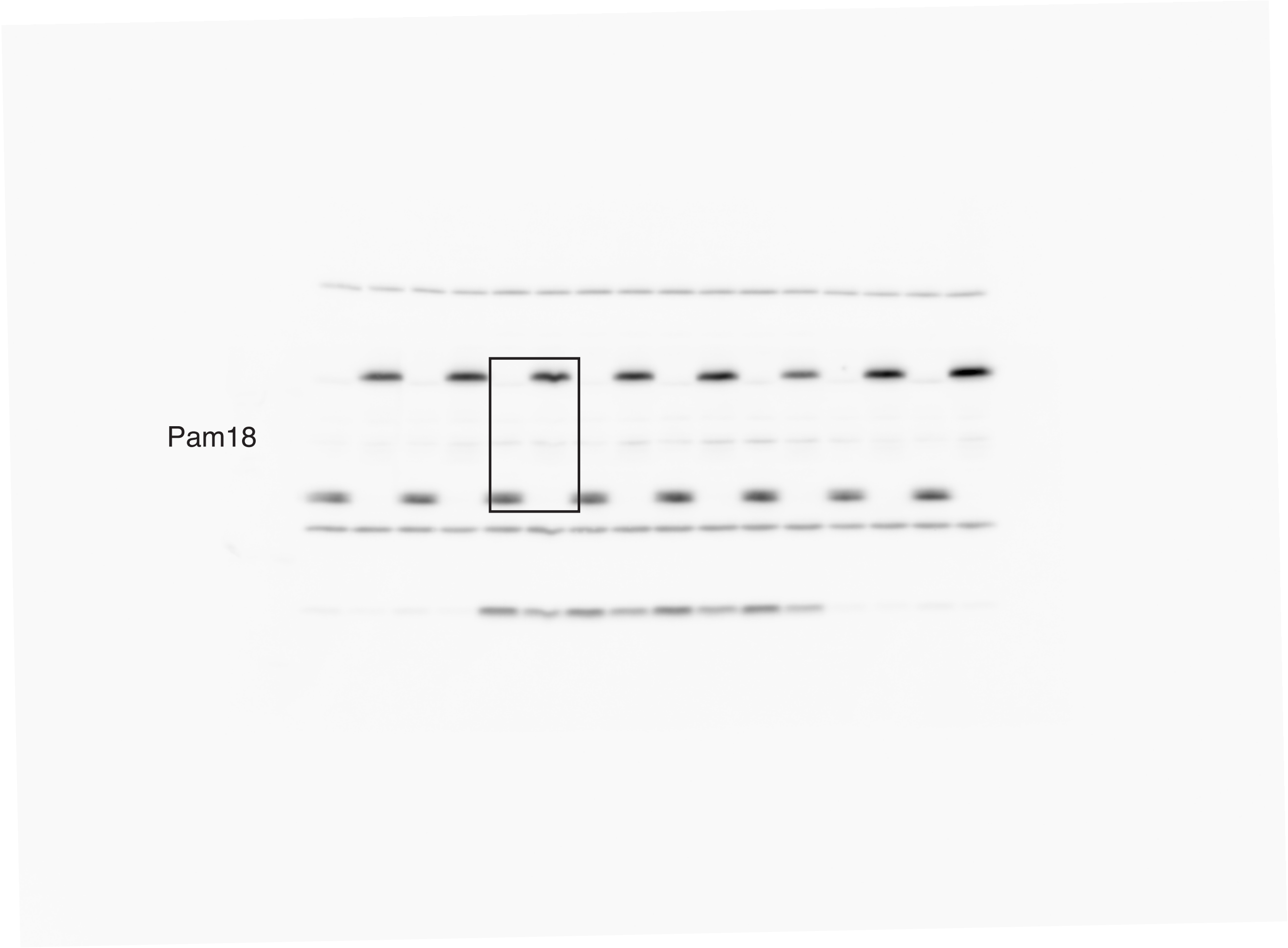

Figure 4a

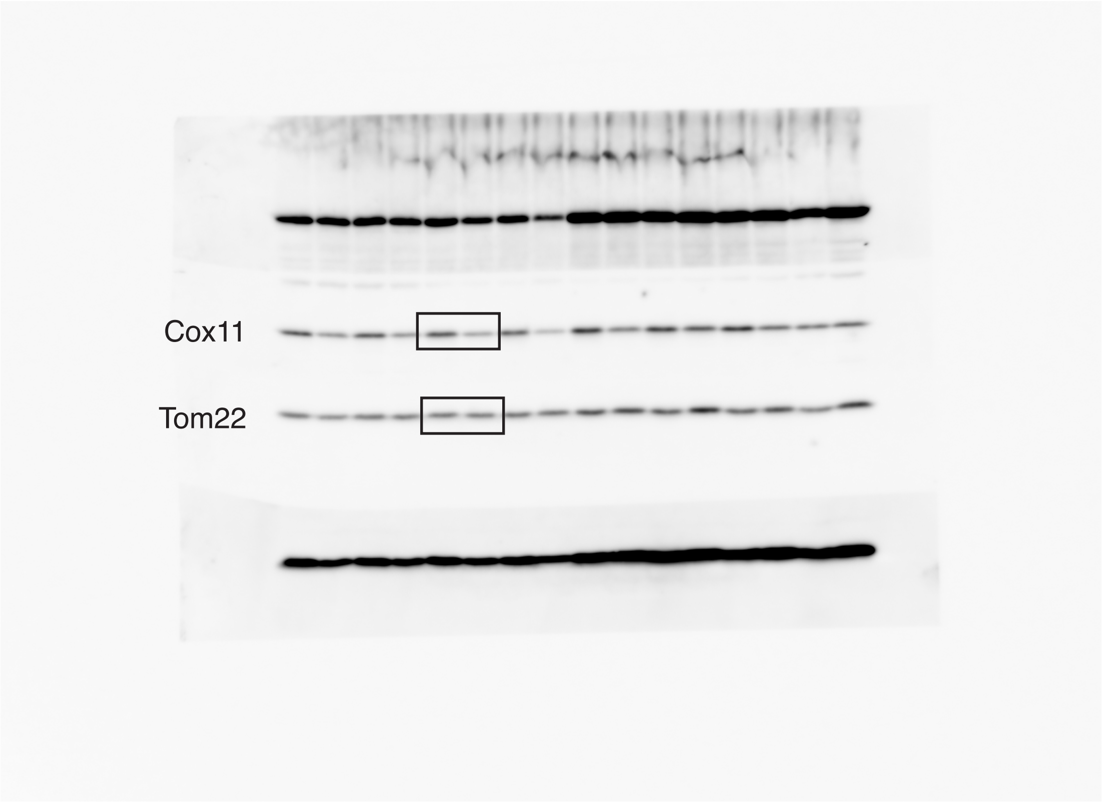

Supplementary Figure 9
Original scans of key Western blots and gels presented in the paper

Figure 4a

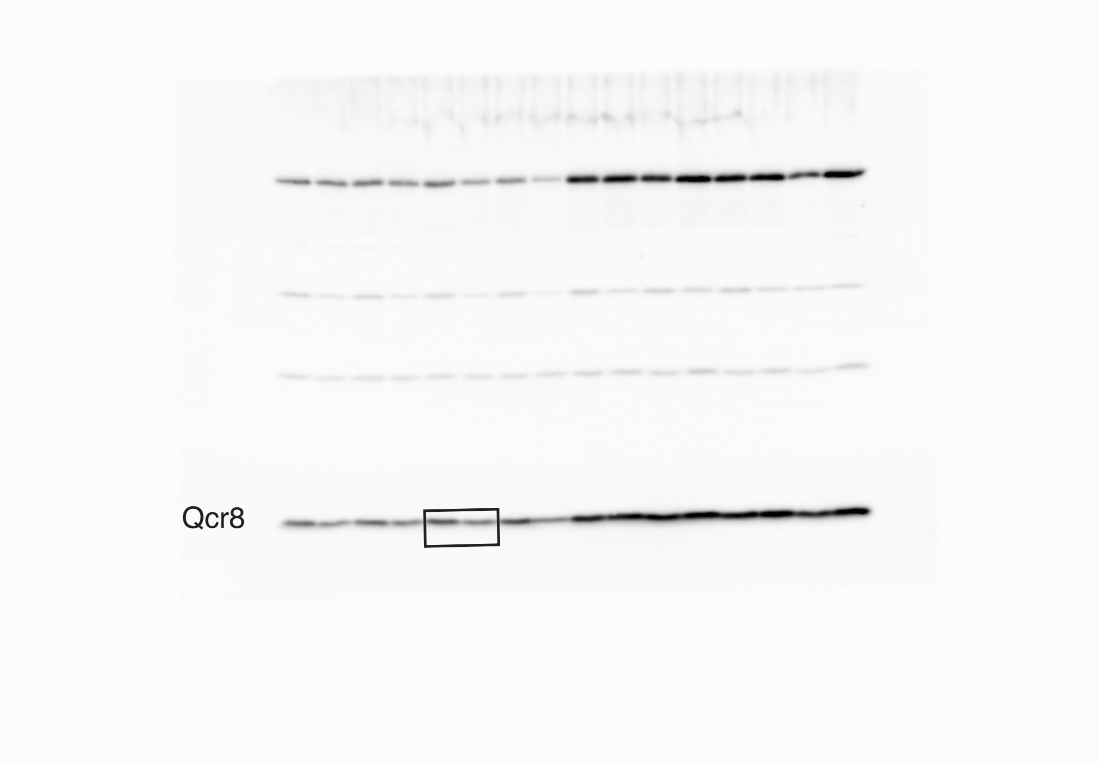

Figure 4a

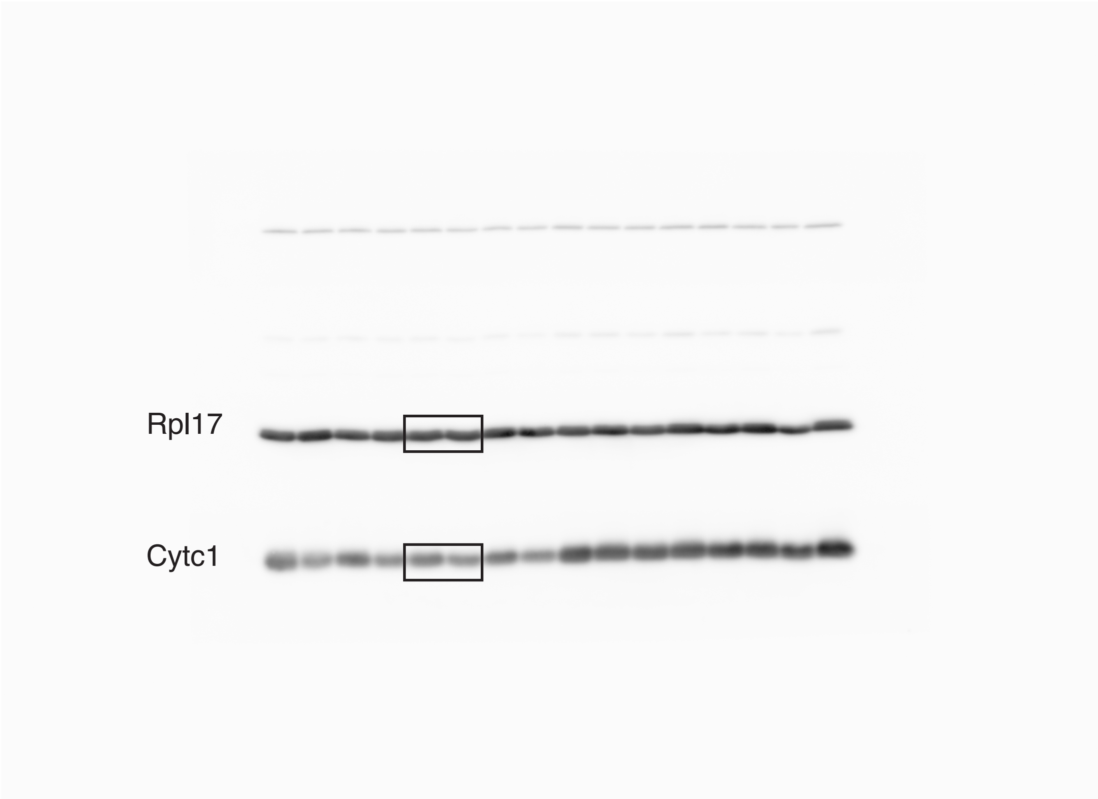

Supplementary Figure 10
Original scans of key Western blots and gels presented in the page

Figure 4a

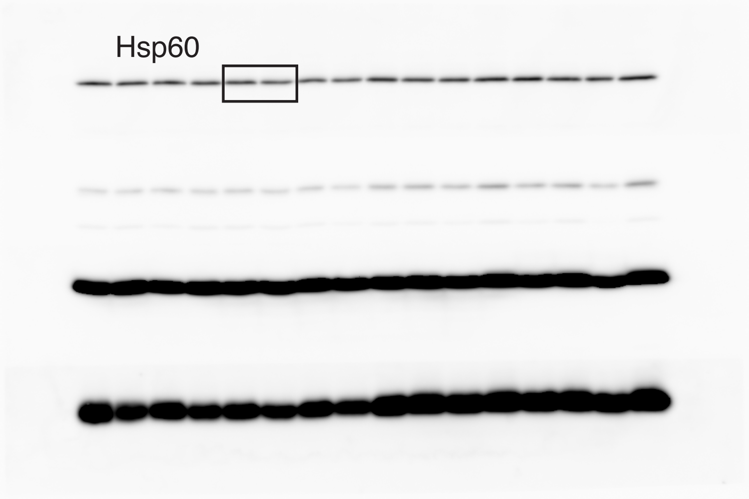

Figure 4c

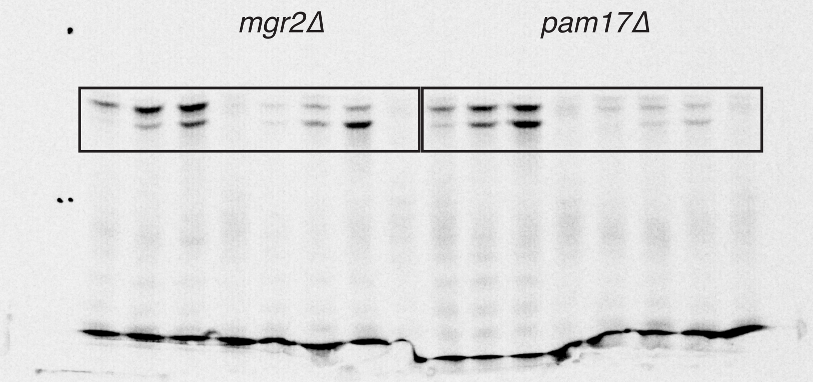

Supplementary Figure 11
Original scans of key Western blots and gels presented in the paper

Figure 4e

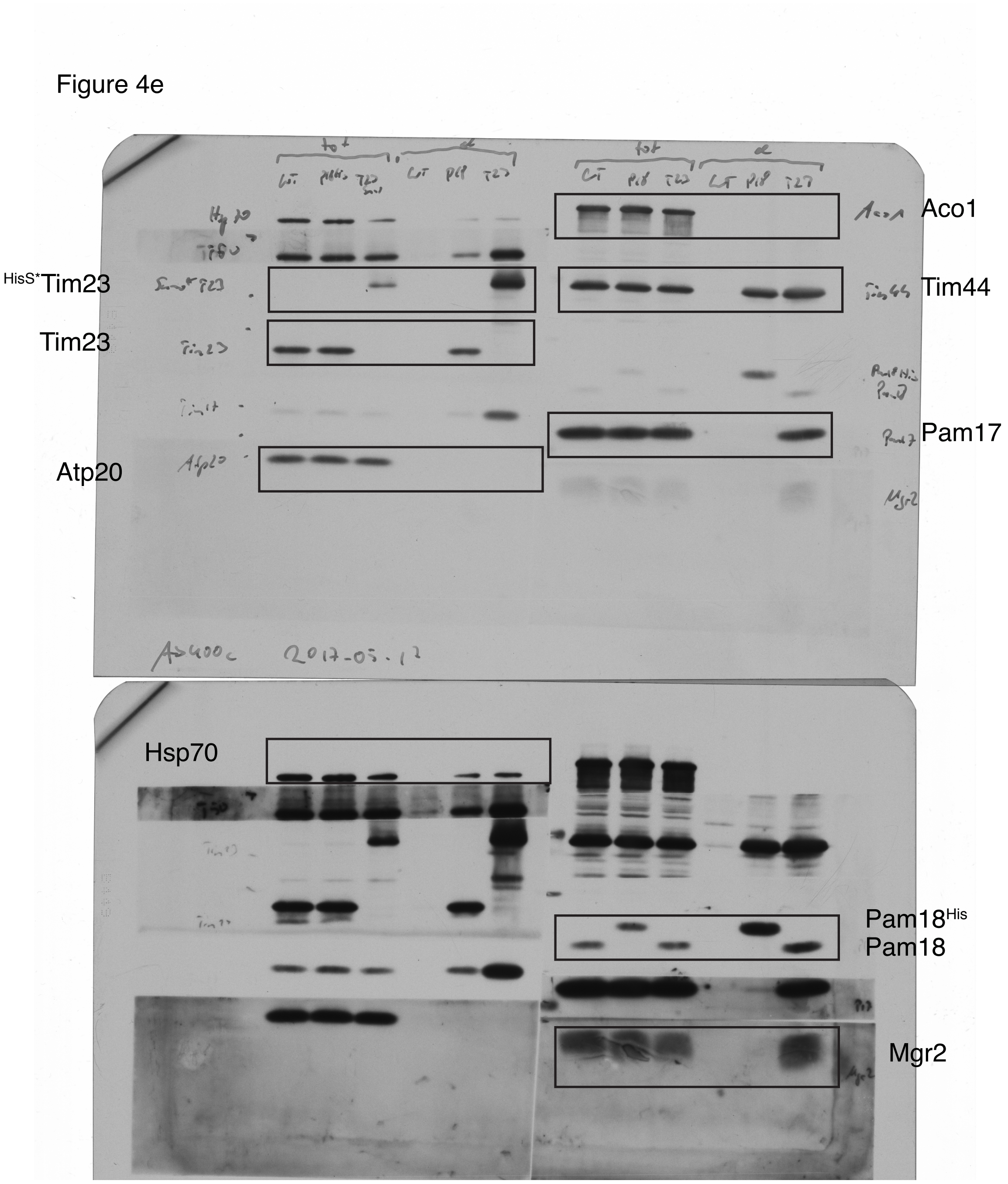

Supplementary Figure 12
Original scans of key Western blots and gels presented in the paper

Figure 4f

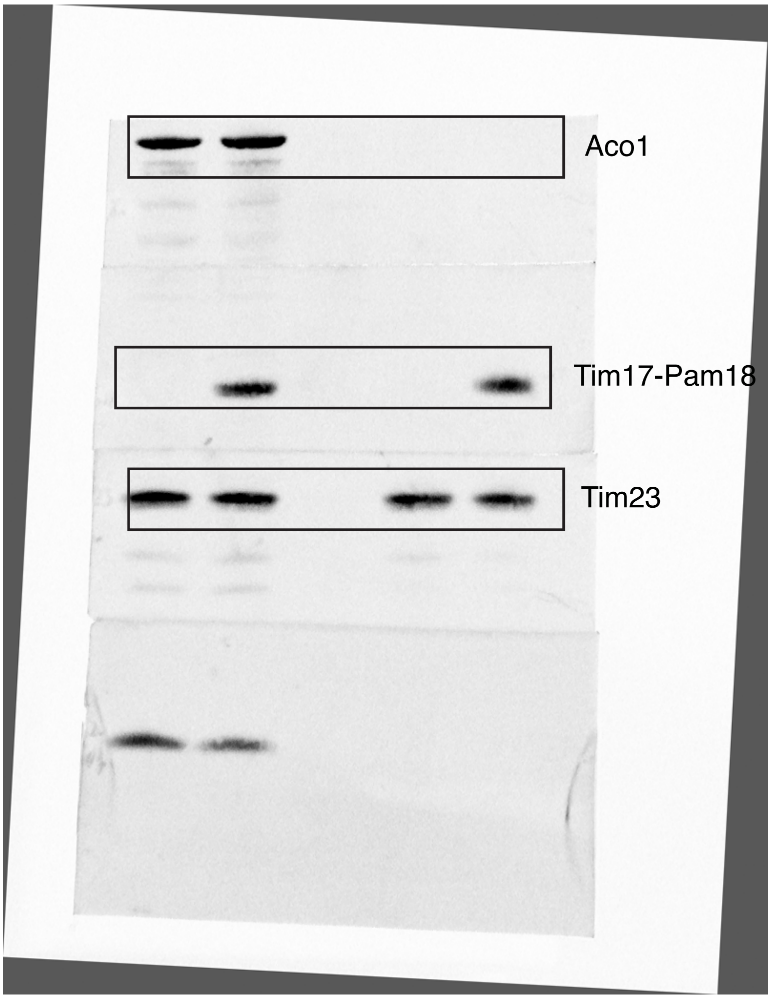

Figure 4f

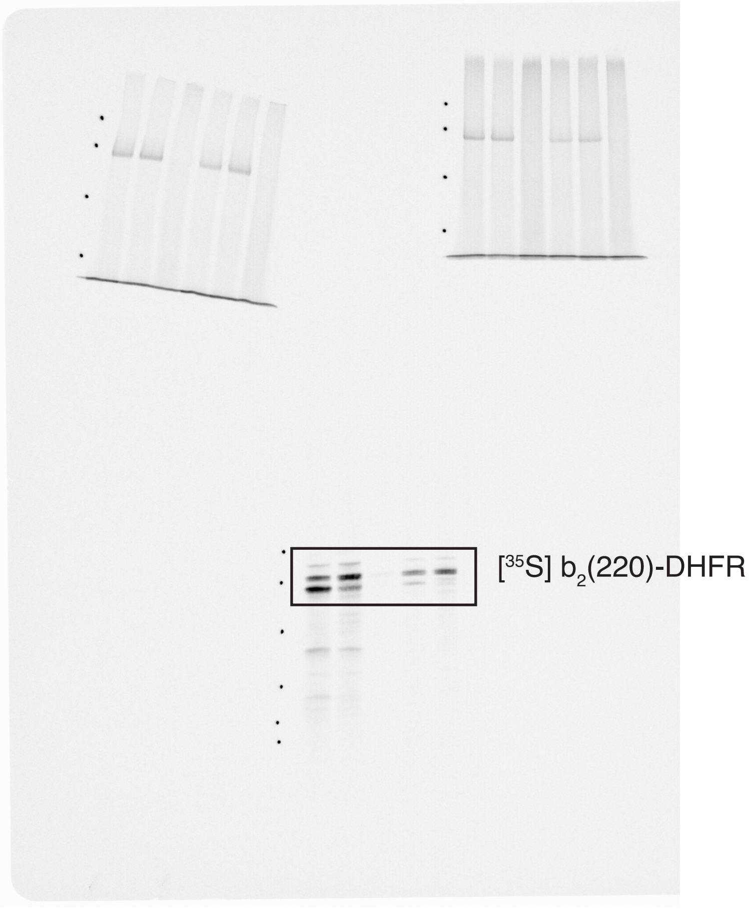

Supplement: Supplementary file 1 — Supplementary Information [file 41467_2018_6492_MOESM1_ESM.pdf]
